# Linear ubiquitination triggers Amph-mediated T-tubule biogenesis

**DOI:** 10.1126/sciadv.ady4934

**Published:** 2026-01-07

**Authors:** Kohei Kawaguchi, Yutaro Hama, Harunori Yoshikawa, Kohei Nishino, Kazuki Morimoto, Tsuyoshi Nakamura, Michiko Koizumi, Yuriko Sakamaki, Kota Abe, Soichiro Kakuta, Koichiro Ichimura, Fumiyo Ikeda, Hidetaka Kosako, Naonobu Fujita

**Affiliations:** ^1^ Cell Biology Center, Institute of Integrated Research, Institute of Science Tokyo, 4259 S2-11 Nagatsuta, Midori-ku, Yokohama 226-8501, Japan.; ^2^ Institute for Genetic Medicine, Hokkaido University, Sapporo 060-0815, Japan.; ^3^ Division of Cell Signaling, Fujii Memorial Institute of Medical Sciences, Institute of Advanced Medical Sciences, Tokushima University, Tokushima 770-8503, Japan.; ^4^ Graduate School of Life Science and Technology, Institute of Science Tokyo, Yokohama 226-8501, Japan.; ^5^ Ochanomizu Research Facility (ORF), Bioscience Center, Research Infrastructure Management Center, Institute of Science Tokyo, Tokyo 113-8510, Japan.; ^6^ Department of Homeostatic Regulation, Division of Cellular and Molecular Biology, Research Institute for Microbial Diseases, The University of Osaka, Suita 565-0871, Japan.; ^7^ Laboratory of Morphology and Image Analysis, Biomedical Research Core Facilities, Juntendo University Graduate School of Medicine, Tokyo 113-8421, Japan.; ^8^ Department of Anatomy and Life Structure, Juntendo University Graduate School of Medicine, Tokyo 113-8421, Japan.; ^9^ Graduate School of Frontier Biosciences, The University of Osaka, Suita 565-0871, Japan.

## Abstract

Transverse tubules (T-tubules) are invaginations of the muscle plasma membrane that facilitate rapid transmission of action potentials, ensuring synchronized muscle contraction. Despite their essential role in muscle physiology, the mechanisms underlying T-tubule formation remain elusive. Here, we identify LUBEL/RNF31, a ubiquitin E3 ligase responsible for linear (M1-linked) ubiquitination, as a key regulator of T-tubule biogenesis in *Drosophila*. Loss of LUBEL leads to Amphiphysin (Amph)–positive membrane sheets instead of tubular networks. The ubiquitin ligase activity of LUBEL and direct interaction with Amph, a BAR domain protein involved in membrane tubulation, are crucial for proper T-tubule morphology. LUBEL and M1-linked ubiquitin chains assemble into puncta on membranes through multivalent interactions, facilitating Amph-mediated tubulation. Notably, the Amph-LUBEL/RNF31 interaction is evolutionarily conserved across species, underscoring a fundamental role for linear ubiquitination in membrane remodeling. Our findings uncover an unexpected function of linear ubiquitination in membrane deformation driven by BAR proteins.

## Teaser

Linear ubiquitination-driven puncta assembly on membranes triggers Amph-mediated T-tubule biogenesis in muscle cells.

## INTRODUCTION

Transverse tubules (T-tubules) are specialized plasma membrane invaginations that form a unique tubular network in muscle cells (*1*). During excitation-contraction coupling, a stimulus from motor neurons triggers depolarization of sarcolemma, the plasma membrane in muscle cells. This depolarization propagates through the T-tubules, ensuring the rapid and synchronized transmission of signals to the sarcoplasmic reticulum (SR), which releases calcium ions into the cytosol, ultimately driving muscle contraction and movement. Defects in T-tubule organization are linked to various congenital muscular diseases, underscoring their physiological significance.

Many key regulators of T-tubule shaping have been identified as causative genes of congenital myopathies (*2*). Centronuclear myopathy (CNM), a severe congenital myopathy characterized by T-tubule defects and muscle weakness, is associated with mutations in three membrane trafficking-related genes: BIN1 (Bridging Integrator-1; also known as Amphiphysin 2, Amph2), DNM2 (Dynamin 2), and MTM1 (Myotubularin 1) (*2*). Amph2 is a BAR domain protein that induces membrane tubulation (*3*–*6*). Its N-terminal BAR domain forms banana-shaped dimers that interact with negatively charged phospholipids via their positively charged concave surfaces. These dimers further assemble into extensive networks, promoting membrane curvature and tubulation (*7*). The C-terminal SH3 domain of Amph2 acts as an adapter, binding to partner proteins such as DNM2, which is a large GTPase (guanosine triphosphatase) involved in membrane scission (*8*). Excessive DNM2 activity disrupts the T-tubule network (*9*), indicating the necessity of precisely regulated DNM2 activity for proper T-tubule formation. The maintenance of the T-tubule network involves MTM1, a phosphatidylinositol 3-phosphate (PI3P) phosphatase (*10*). In addition, the muscle-specific caveolae-forming protein Cav3 has been implicated in the early stage of T-tubule formation and is associated with several myopathies, including limb-girdle muscular dystrophy (LGMD1C). The caveolin-associating coat protein, Cavin4, is also required for T-tubule formation and has been identified as a causative gene for dilated cardiomyopathy (*11*, *12*). Moreover, its paralog, Cavin1, has been shown to contribute to T-tubule formation in mice and zebrafish (*13*). Cavin4 has recently been reported to bridge caveolae and Amph2, forming a ringlike platform at the sarcolemma that acts as a scaffold for initiation of T-tubule formation (*11*, *12*). However, although the caveola-forming caveolins Cav1 and Cav3 are conserved in chordates and vertebrates, they are absent in other metazoans (*14*). T-tubules emerged evolutionarily before the caveolin-mediated membrane deformation mechanisms (*15*–*17*), suggesting the existence of an alternative: a caveolin-independent mechanism for T-tubule biogenesis.

*Drosophila* has emerged as an ideal model organism for studying T-tubule biogenesis. Several characteristics make *Drosophila* particularly suited for T-tubule research: First, T-tubules are not essential for viability in flies (*5*); second, T-tubules can be observed through the cuticle in live animals; and third, *Drosophila* is genetically tractable. These advantages enable reverse genetic analysis, which is essential for elucidating mechanisms of T-tubule formation. This study used proximity labeling, an enzymatic reaction that enables the identification of local proteomes, and in vivo RNA interference (RNAi) screening to identify key T-tubule regulators. Our analysis revealed that LUBEL, an E3 ligase responsible for linear (M1-linked) ubiquitination, plays a crucial role in T-tubule biogenesis. LUBEL promotes the formation of M1-linked ubiquitin chains, in which the α-amino group of the N-terminal methionine (M1) of ubiquitin is used instead of the ε-amino group of lysine residues (K6, K11, K27, K29, K33, K48, and K63) (*18*–*20*). Further mechanistic investigations into LUBEL uncovered an unexpected role of linear ubiquitination in BAR protein–mediated membrane remodeling.

## RESULTS

### Proximity proteomics of T-tubules

**Fig. 1.**
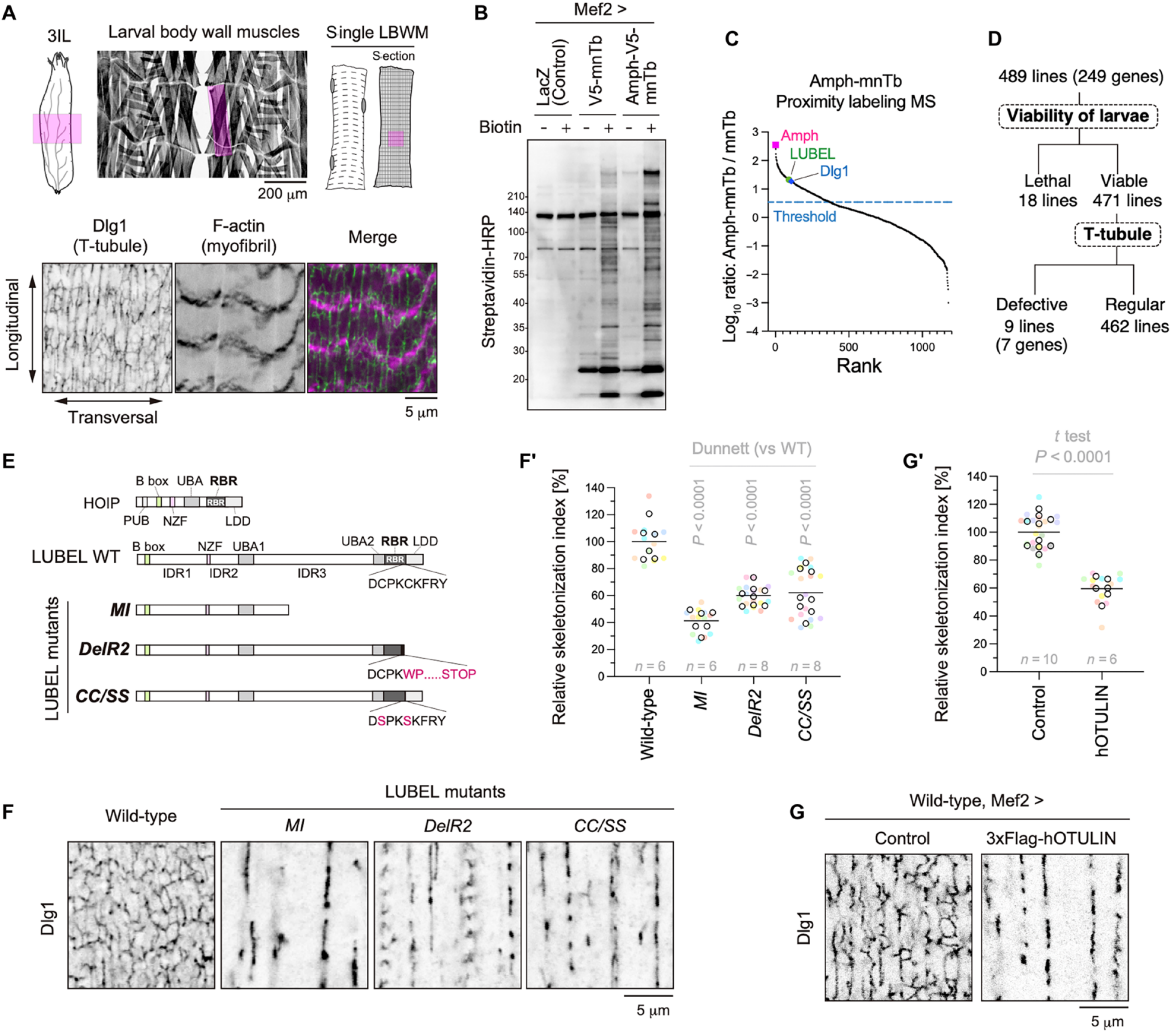
Proximity proteomics and RNAi screening identify LUBEL as a critical regulator of T-tubule formation. (**A**) T-tubules in the 3IL BWM. Representative images of T-tubules (Dlg1, green) and myofibrils (F-actin, magenta) in the midsection of 3IL BWMs. (**B**) Streptavidin blotting of larval carcass fillet lysates. LacZ, mnTb, or Amph-mnTb was expressed in BWMs. Larvae were reared with or without biotin for 2 days before sample preparation. (**C**) Proximity proteomics of T-tubules using Amph-mnTb. The *y* axis represents the fold change (Amph-mnTb versus mnTb control), whereas the *x* axis indicates the rank. (**D**) Summary of RNAi screening results. (**E**) Schematic representation of LUBEL mutants. LUBEL contains the indicated domains. LUBEL mutants either lack the C-terminal region or harbor specific mutations. (**F** and **F′**) T-tubules in 3IL BWMs of indicated genotypes. Images of anti-Dlg1 staining (F) and quantification of skeletonized Dlg1-positive structures (F′). (**G** and **G′**) Effect of human OTULIN overexpression on T-tubules. Images of anti-Dlg1 staining (G) and quantification of skeletonized Dlg1-positive structures (G′).

We previously established an imaging-based RNAi screening system for T-tubules in *Drosophila* larval body wall muscles (BWMs), which have developed T-tubules oriented both longitudinally and transversely (Fig. 1A) (*21*). Although genome-wide RNAi screening is feasible in *Drosophila*, it is time-intensive and labor-intensive. To identify candidate genes unbiasedly, we used biotinylation-based proximity proteomics of T-tubules in vivo. We generated a transgenic line carrying an Amph construct fused with miniTurbo (mnTb), an engineered biotin ligase designed for proximity labeling (*22*). Immunostaining confirmed that Amph-mnTb, but not mnTb alone, colocalized with Dlg1, an established T-tubule marker, in larval muscle cells (fig. S1A), in the same manner as endogenous Amph (*5*, *21*).

To verify the biotinylation capability of Amph-mnTb, we expressed either Amph-mnTb or mnTb alone in muscles, and larvae were reared in the presence or absence of biotin. As a control, LacZ was expressed instead of the mnTb-fused constructs. Streptavidin–horseradish peroxidase (HRP) blotting of larval carcass lysates revealed intense biotin-dependent labeling in Amph-mnTb and mnTb samples but not in controls (LacZ) (Fig. 1B). Notably, distinct banding patterns between Amph-mnTb and mnTb samples were observed under biotin-feeding conditions, suggesting that Amph-mnTb biotinylates its proximal proteins. Biotinylated peptides were purified from trypsin-digested samples using Tamavidin2-REV beads and analyzed by mass spectrometry (MS) (*23*), revealing the proximity proteome of Amph in muscle cells (data S1). As expected, Dlg1 was substantially enriched in Amph-mnTb samples (Fig. 1C and data S1), confirming successful T-tubule proximity labeling.

### Muscle-targeted RNAi screening for T-tubule regulators

From the proximity proteomics data (data S1), we selected 249 genes surpassing the enrichment threshold for muscle-targeted RNAi screening for T-tubule regulators (Fig. 1D). A total of 489 UAS-RNAi lines were crossed with a line carrying both Dlg1–green fluorescent protein (GFP), a T-tubule marker driven by its endogenous promoter, and Mef2-GAL4. Of these, 18 RNAi lines resulted in lethality; thus, the remaining 471 viable lines were further analyzed. Confocal microscopy imaging of Dlg1-GFP–labeled T-tubules in intact larvae identified nine RNAi lines exhibiting defects in T-tubule morphology (Fig. 1D and data S2).

Among them, three independent RNAi lines targeting LUBEL induced the most severe phenotype, resembling the T-tubule defects observed in Amph RNAi (fig. S1B). Both conditions resulted in the loss of transverse and thick longitudinal membranes labeled with Dlg1. LUBEL is the *Drosophila* ortholog of HOIP, the catalytic subunit of the linear ubiquitin chain assembly complex (LUBAC) in mammals (*19*). LUBAC is composed of three subunits: HOIP (HOIL-1–interacting protein), HOIL-1 (heme-oxidized IRP2 ubiquitin ligase 1), and SHARPIN (SHANK-associated RH domain–interacting protein). Among these, HOIP acts as the catalytic subunit responsible for M1-linked ubiquitination, whereas HOIL-1 and SHARPIN function as regulatory subunits that stabilize and regulate LUBAC activity (*24*–*28*). Unlike its mammalian counterpart, *Drosophila* LUBEL is sufficient for M1-linked ubiquitination without additional regulatory subunits (*19*). LUBEL shares multiple conserved domains with HOIP, including the B-box, Npl4 Zinc Finger (NZF), ubiquitin-associated (UBA), RING-between-RING (RBR), and linear ubiquitin determinant (LDD) domains (Fig. 1E). In addition, unlike HOIP, LUBEL contains extended intrinsically disordered regions (IDRs) between key domains (*19*), contributing to its larger size (2902 amino acids versus 1072 amino acids in human HOIP). Although M1-linked ubiquitination is critical for immune signaling pathways via nuclear factor κB (NF-κB), its role in membrane remodeling remains unexplored. Given the pronounced T-tubule defects observed upon LUBEL knockdown (fig. S1B), we focused on its function in subsequent analyses.

### M1-linked ubiquitination by LUBEL is indispensable for T-tubule morphology

To validate the RNAi-based knockdown phenotype, we analyzed a genomic mutant, *LUBEL^MI^*, which harbors a stop codon in the middle of the protein, leading to the loss of the entire C-terminal region, including the RBR domain (Fig. 1E). This mutant exhibited severe T-tubule defects (Fig. 1, F and F′, and fig. S2), confirming that LUBEL is essential for T-tubule formation. Next, we assessed whether the E3 ligase activity of LUBEL was required for T-tubule formation by analyzing mutant strains carrying enzymatically inactive variants (Fig. 1E). The *LUBEL^DelR2^* mutant, with a truncation of the C-terminal region of the RBR, and the *LUBEL^CC/SS^* mutant, carrying mutations in essential cysteine residues, both displayed abnormal T-tubule morphology (Fig. 1F), demonstrating that the E3 ligase activity of LUBEL is critical for T-tubule formation.

To determine whether M1-linked ubiquitination, rather than other ubiquitin linkages, is essential for T-tubule formation, we overexpressed human OTULIN, a deubiquitinating enzyme specific for M1-linked ubiquitin chains (*29*), in wild-type *Drosophila* muscle cells. Notably, OTULIN overexpression recapitulated the T-tubule defects observed in LUBEL loss-of-function mutants (Fig. 1, G and G′), indicating an essential role of M1-linked ubiquitin chains in T-tubule formation. Together, these findings demonstrate that LUBEL-mediated linear ubiquitination is crucial for T-tubule formation.

### T-tubule formation is independent of the NF-κB signaling pathway

LUBAC-mediated M1-linked ubiquitination is well established as a key regulator of the NF-κB signaling pathway in vertebrates (*20*, *30*). LUBAC ubiquitinates NEMO (NF-κB essential modulator), a regulatory subunit of the IKK (IκB kinase) complex, leading to the activation of NF-κB transcription factors (*24*–*26*, *28*). The role of M1-liked ubiquitination in NF-κB signaling is also evolutionarily conserved in *Drosophila* (*31*). To investigate whether the NF-κB signaling pathway is required for T-tubule formation, we analyzed T-tubule morphology in loss-of-function mutants of this pathway. *Rel* encodes the *Drosophila* NF-κB protein, and *Kenny* is the *Drosophila* ortholog of IKKγ/NEMO (*32*, *33*). Both loss-of-function mutants, *Rel[E20]* and *Kenny[c02831]*, exhibited normal T-tubule morphology (fig. S3), suggesting that NF-κB signaling is dispensable for T-tubule formation.

### LUBEL plays a critical role in membrane tubule extension mediated by Amph

**Fig. 2.**
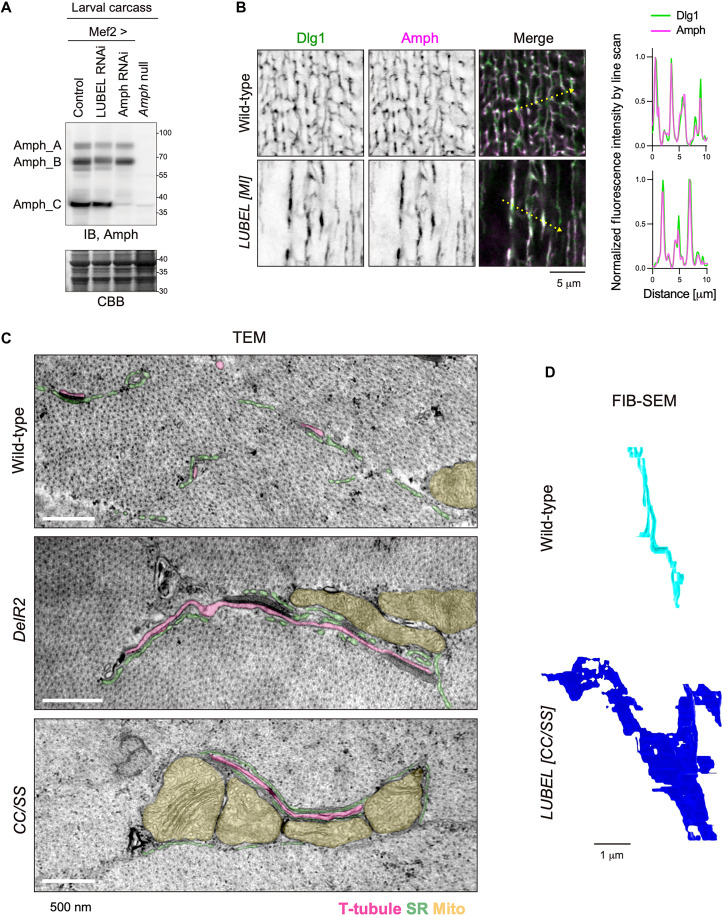
LUBEL is essential for Amph-dependent membrane tubulation. (**A**) Amph immunoblot of 3IL carcass fillet lysates from the indicated genotypes. Coomassie brilliant blue (CBB) staining was used as a loading control. IB, immunoblotting. (**B**) Immunostaining of Amph (magenta) and Dlg1(green) in wild-type and LUBEL mutant 3IL BWMs. Line plot profiles along the yellow dotted arrow are shown. (**C**) TEM images of wild-type and LUBEL mutant 3IL BWMs. T-tubules (magenta), SR (green), and mitochondria (yellow) are highlighted. (**D**) 3D reconstruction models generated by FIB-SEM. T-tubules in wild-type (cyan) and T-tubule–related membranes in LUBEL mutant (blue) are shown.

Amph shapes tubular morphology through its membrane curvature activity (*3*–*6*). On the basis of the evidence that loss of LUBEL phenocopies Amph RNAi (fig. S1B), we hypothesized that loss of LUBEL leads to a decrease in Amph protein levels, resulting in T-tubule defects. To test this, we performed immunoblotting of larval carcass lysates using anti-Amph antibodies (Fig. 2A). In the control sample, three Amph splicing isoforms—A, B, and C—were detected. Muscle-targeted Amph knockdown using Mef2-GAL4 specifically reduced Amph_C levels without affecting isoforms A or B, indicating that Amph_C is predominantly expressed in larval muscle cells. Contrary to our assumption, LUBEL knockdown did not alter Amph_C protein levels (Fig. 2A). These results indicate that the T-tubule defect caused by LUBEL deficiency is not due to changes in Amph protein levels.

Next, we investigated whether LUBEL influences Amph membrane localization by performing immunostaining for endogenous Amph in wild-type and LUBEL mutant muscle cells. As previously reported, Amph colocalized with Dlg1 on T-tubules in wild-type cells (Fig. 2B) (*5*, *21*). Although we observed altered Dlg1-positive membrane morphology, Amph and Dlg1 remained colocalized in LUBEL mutant muscle cells (Fig. 2B). This suggests that LUBEL does not affect the membrane localization of Amph but may regulate its membrane-bending function.

Although confocal microscopy revealed defects in Dlg1-positive membranes in LUBEL mutants, their ultrastructure remained unclear. To explore their ultrastructure, we performed transmission electron microscopy (TEM) imaging of cross sections of larval muscle cells from wild-type and LUBEL mutants (Fig. 2C). In wild-type muscle cells, circular T-tubule cross sections (highlighted in pink) were frequently observed. In contrast, LUBEL mutants exhibited elongated, tubelike structures (pink), which may have originated from T-tubules or their precursors. In addition, these tubular structures were often flanked by the SR (green) and mitochondria (yellow), suggesting the importance of T-tubules in the spatial organization of the SR and mitochondria within muscle cells.

To further elucidate the three-dimensional (3D) architecture of T-tubule–related membranes in LUBEL mutants, we conducted focused ion beam scanning electron microscopy (FIB-SEM). T-tubules and their derived membranes were segmented in individual SEM images and reconstructed into 3D models. In wild-type muscle cells, T-tubules exhibited a tubular morphology, as expected. In contrast, T-tubule–related membranes in LUBEL mutants appeared as flat, sheetlike structures (Fig. 2D and movies S1 and S2). These findings collectively indicate that LUBEL promotes membrane tubule extension without affecting Amph protein levels.

### LUBEL directly interacts with Amph to form T-tubules

**Fig. 3.**
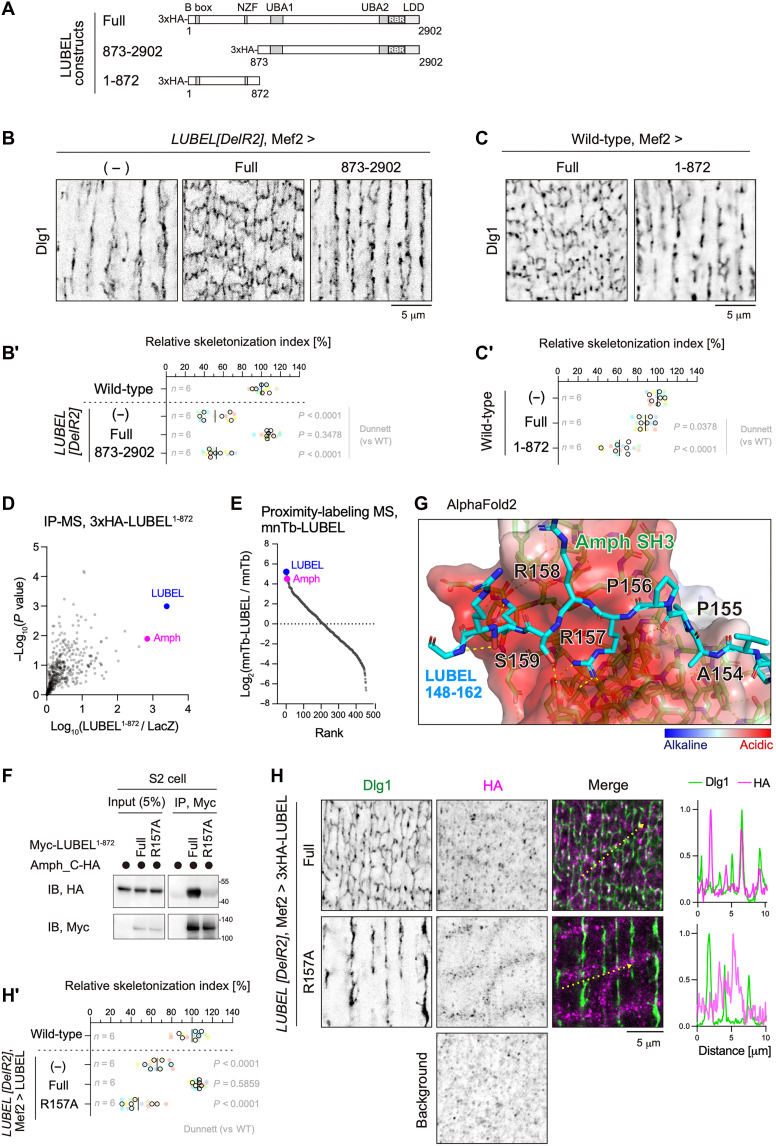
LUBEL-Amph interaction is essential for T-tubule formation. (**A**) Schematic representation of LUBEL truncations. (**B** and **B′**) LUBEL rescue experiment with full-length protein (Full) or a construct lacking the N-terminal region (873 to 2902). 3IL BWMs were stained for Dlg1 (B), and their relative skeletonization index was quantified (B′). (**C** and **C′**) Effect of the LUBEL N-terminal fragment overexpression on T-tubules. Full-length protein (Full) or its N-terminal fragment (1 to 872) was expressed in wild-type 3IL BWMs, followed by staining for Dlg1 (C). HA immunostaining images are shown in fig. S4A. The relative skeletonization index is shown (C′). (**D**) IP-MS analysis using HA-LUBEL^1-872^. HA-LUBEL^1-872^ or LacZ (control) was expressed in 3IL BWMs. Lysates from carcass fillets were subjected to anti-HA IP and analyzed by LC-MS/MS. The *x* axis shows the fold change (HA-LUBEL^1-872^ versus LacZ control), whereas the *y* axis represents the negative log_10_ *P* value from two replicates. (**E**) Proximity proteomics using mnTb-LUBEL. The *y* axis represents the fold change (mnTb-LUBEL versus mnTb control), whereas the *x* axis indicates the rank. (**F**) Co-IP assay of Amph and LUBEL^R157A^. Myc-LUBEL^1-872^ (wild-type or R157A mutant) and Amph isoform C-HA were coexpressed in S2 cells. Lysates were subjected to anti-Myc IP and immunoblotted for anti-Myc and anti-HA antibodies. (**G**) Structure of the Amph SH3 and LUBEL-N fragment complex using AlphaFold2. For LUBEL, only residues 148 to 168 are displayed. (**H** and **H′**) LUBEL rescue experiments using full-length protein (Full) or the LUBEL^R157A^ (R157A) mutant. 3IL BWMs were stained with anti-Dlg1 and anti-HA antibodies (H). The relative skeletonization index is shown (H′).

LUBEL contains multiple domains besides the RBR domain (Fig. 3A). To assess whether its N-terminal region contributes to T-tubule biogenesis, we performed rescue experiments using either a full-length LUBEL construct (Full) or one lacking the N-terminal region (LUBEL^873-2902^) (Fig. 3A). The full-length construct rescued the T-tubule defects in the *LUBEL^DelR2^* mutant, whereas LUBEL^873-2902^ did not (Fig. 3, B and B′). Moreover, overexpression of the N-terminal fragment (LUBEL^1-872^) disrupted the T-tubule morphology even in wild-type muscle cells (Fig. 3, C and C′). Furthermore, LUBEL^1-872^ localized to Dlg1-positive membranes (fig. S4A), suggesting that the N-terminal region may sequester an unknown factor required for membrane localization. To identify potential binding partners, we performed immunoprecipitation–mass spectrometry (IP-MS) of LUBEL^1-872^ (Fig. 3D and data S3) and proximity proteomics using mnTb-fused LUBEL (Fig. 3E and data S4). Both analyses consistently identified Amph as a high-confidence candidate interactor of LUBEL.

To map the LUBEL-binding region in Amph, we performed glutathione *S*-transferase (GST) pull-down assays using truncated Amph constructs (fig. S4B). The C-terminal SH3 domain, but not the N-terminal BAR domain, pulled down LUBEL^1-872^ (fig. S4C), indicating that the SH3 domain mediates the interaction between LUBEL and Amph. In silico analysis using AlphaFold2 predicted that four proline-rich motifs and the B-box zinc finger domain in LUBEL^1-872^ are binding sites for the Amph SH3 domain (*34*) (fig. S4D). To validate this, we generated LUBEL point mutants designed to disrupt these interactions (fig. S4E) and performed coimmunoprecipitation (co-IP) assays with Amph (fig. S4E′). The LUBEL R157A mutation, an alanine substitution of Arg^157^, abolished binding with Amph (Fig. 3F and fig. S4E′), identifying the proline-rich motif (152 to 157) as the binding site for the Amph SH3 domain. AlphaFold2 multimer modeling predicted that LUBEL Arg^157^ is buried in a 139.8-Å^2^ surface area of the Amph SH3 domain, which is 29.6% of the total binding interface (472.0 Å^2^). Arg^157^ is positioned to bind within an acidic pocket of the Amph SH3 domain with three glutamate and aspartate residues and forms several salt bridges and hydrogen bonds with these acidic residues (Fig. 3G). These results suggest that the electrostatic interactions between the Arg^157^-containing proline-rich motif and the SH3 domain are important for LUBEL-Amph interaction.

To assess the functional significance of the LUBEL-Amph interaction in T-tubule formation, we expressed the LUBEL^R157A^ in LUBEL-deficient muscle cells. Unlike the full-length protein, LUBEL^R157A^ failed to rescue T-tubule defects (Fig. 3, H and H′). Moreover, LUBEL R157A did not colocalize with Dlg1 (Fig. 3H). These findings highlight the critical role of the interaction between LUBEL and Amph in T-tubule formation.

### LUBEL and M1-linked ubiquitin chains form puncta on T-tubules

**Fig. 4.**
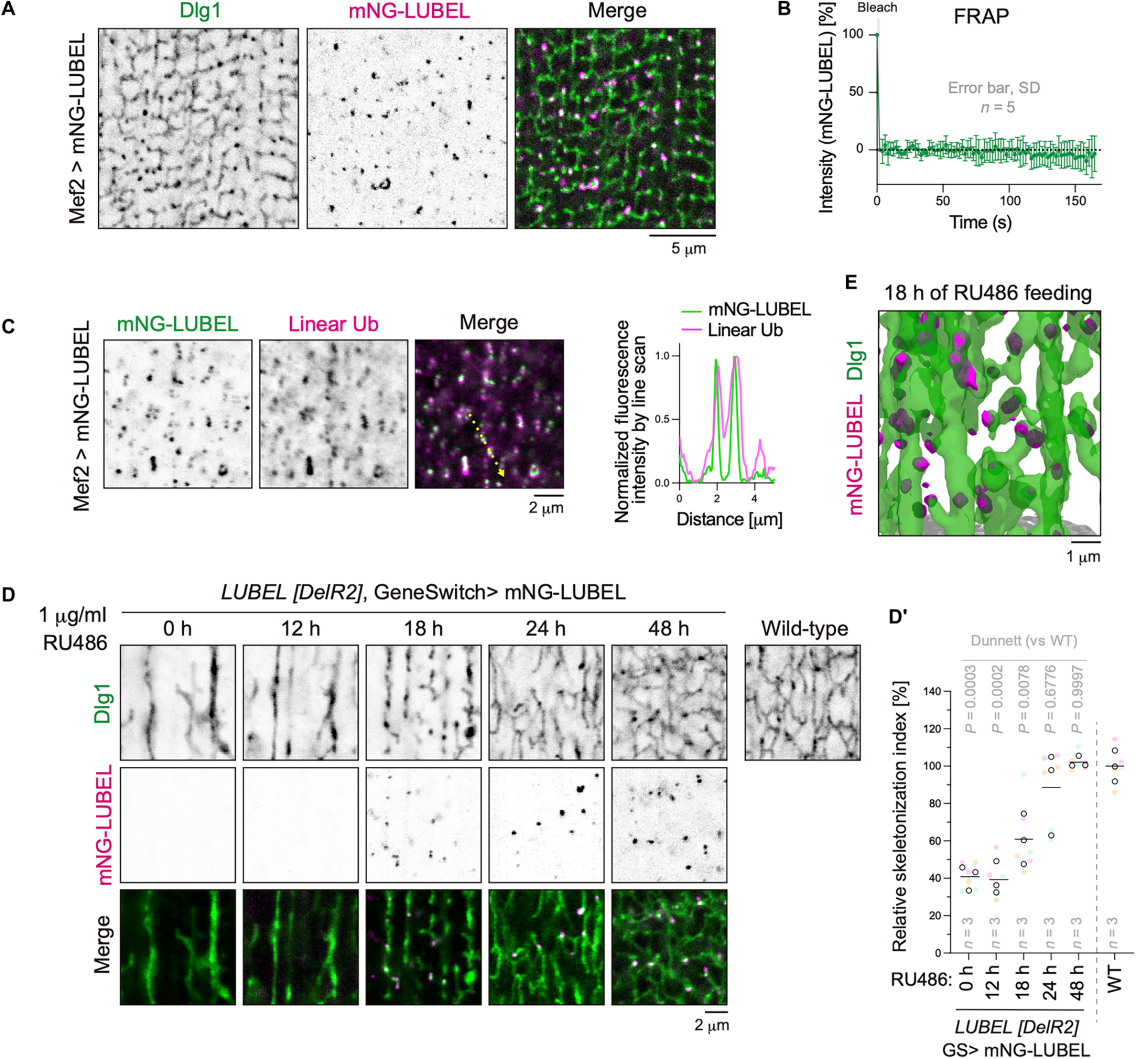
LUBEL and M1-linked ubiquitin chains form puncta on T-tubules. (**A**) Localization of mNG-LUBEL on T-tubules. 3IL BWMs expressing mNG-LUBEL were stained with anti-Dlg1 antibody. (**B**) FRAP analysis of mNG-LUBEL in 3IL BWMs. (**C**) Colocalization of mNG-LUBEL and M1-linked ubiquitin chains. 3IL BWMs expressing mNG-LUBEL were stained with anti-Dlg1 and anti–linear ubiquitin antibodies. (**D** and **D′**) LUBEL rescue experiment with temporally controlled mNG-LUBEL expression. mNG-LUBEL expression was regulated using the GeneSwitch system. Larvae were reared on RU486-containing food for the indicated duration before reaching the late 3IL stage. 3IL BWMs were stained with anti-Dlg1 antibody (D), and their relative skeletonization index was quantified (D′). h, hours. (**E**) 3D reconstitution of *z*-series fluorescence images of mNG-LUBEL and Dlg1 in 3IL BWMs after 18 hours of RU486 feeding.

To examine the subcellular localization of LUBEL, we expressed mNeonGreen (mNG)–tagged LUBEL in wild-type muscle cells. mNG-LUBEL did not exhibit tubular network morphology but formed punctate structures that colocalized with Dlg1 and Amph (Fig. 4A and fig. S5). To determine whether the expression level of LUBEL affects its localization, the GeneSwitch GAL4 (GS) system was used (*35*). Feeding larvae with RU486 (also known as mifepristone), an inducer of the GS system, triggers mNG-LUBEL expression in a concentration-dependent manner. These LUBEL-positive puncta were also observed in live muscle cells at expression levels comparable to endogenous LUBEL (fig. S6), excluding the possibility that they are artifacts caused by overexpression or fixation. To evaluate the dynamic properties of LUBEL puncta, we performed fluorescence recovery after photobleaching (FRAP) analysis in live larval BWM cells expressing mNG-LUBEL. Following photobleaching, no substantial fluorescence recovery was observed (Fig. 4B and fig. S7), indicating that LUBEL forms puncta with low fluidity. Furthermore, the LUBEL puncta also contained M1-linked ubiquitin chains (Fig. 4C). Given its localization and role in membrane tubulation, LUBEL likely localizes to specific sites during T-tubule formation. However, direct observation of T-tubule formation is challenging due to its concurrence with myofibril differentiation. To overcome this issue, we used the GS system to control mNG-LUBEL expression in LUBEL mutants temporally (*35*). In the absence of RU486, Dlg1 appeared in thick, sheetlike structures (Fig. 4D). After 48 hours of RU486 feeding, T-tubule defects in LUBEL mutants were almost completely rescued (Fig. 4, D and D′), indicating that Dlg1-positive sheets transitioned into tubular structures upon mNG-LUBEL expression. Partial recovery was observed after 18 and 24 hours of RU486 feeding, suggesting that Dlg1-positive structures are forming T-tubules. Notably, mNG-LUBEL puncta were observed at the edge of these Dlg1-positive T-tubule intermediates. A 3D reconstruction of *z*-series images (Fig. 4E) revealed that LUBEL puncta were frequently located at protrusions of forming T-tubules, suggesting a role in the early stage of T-tubule biogenesis.

### Attractive and repulsive interactions among LUBEL puncta

**Fig. 5.**
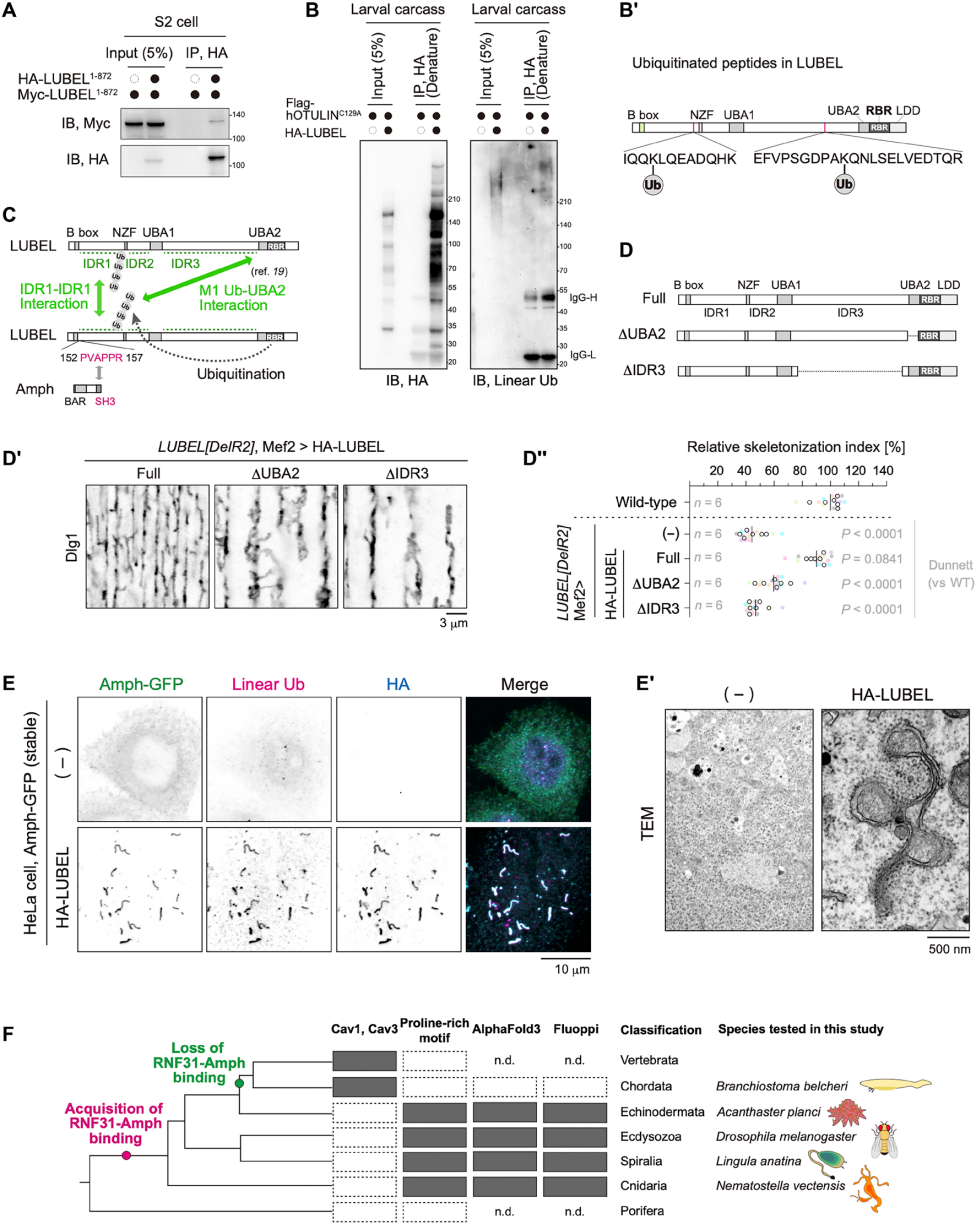
LUBEL cluster and membrane deformation. (**A**) Co-IP assay of the LUBEL N-terminal fragment. HA-LUBEL^1-872^ and Myc-LUBEL^1-872^ were coexpressed in S2 cells. Lysates were subjected to anti-HA IP and immunoblotted for the indicated antibodies. (**B**) LUBEL autoubiquitination. HA-LUBEL and the catalytically dead human OTULIN^C129A^ mutant were coexpressed in 3IL BWMs. Carcass fillets were lysed in SDS-containing denaturing buffer, diluted 10-fold, and subjected to anti-HA IP. Samples were then immunoblotted with the indicated antibodies. (**B′**) Ubiquitination sites of LUBEL. The corresponding MS/MS spectra of the diglycine-modified peptides are presented in fig. S9. (**C**) Schematic representation of LUBEL-mediated interactions. LUBEL potentially self-associates through IDRs and undergoes intra- or intermolecular autoubiquitination. The UBA2 domain has an affinity for M1-linked ubiquitin chains. (**D**, **D′**, and **D″**) LUBEL rescue experiment using full-length (Full), ΔUBA2, or ΔIDR3 constructs (D). 3IL BWMs were stained with anti-Dlg1 antibody (D′), and the relative skeletonization index was quantified (D″). (**E** and **E′**) LUBEL-dependent membrane tubulation in HeLa cells. HeLa cells stably expressing Amph-GFP were transfected with either HA-LUBEL or an empty vector and stained with the indicated antibodies (E) or analyzed by TEM (E′). (**F**) Evolutionary analysis of LUBEL-Amph interactions across animal species. For further details, see figs. S10 and S11. n.d., not determined.

LUBEL and M1-linked ubiquitin chains form puncta on membranes, possibly through multivalent interactions. Because LUBEL contains long IDRs, which can facilitate weak intra- and intermolecular interactions, we investigated whether LUBEL undergoes homotypic interactions. Myc-LUBEL^1-872^ was coimmunoprecipitated with HA-LUBEL^1-872^ (Fig. 5A). Furthermore, IP-MS of LUBEL^1-872^ identified LUBEL fragments corresponding to its C-terminal half (Fig. 3D and data S3). These findings suggest that LUBEL interacts with itself intermolecularly through its IDR1 (Fig. 5C). Of note, in silico analysis predicted that LUBEL harbors both attractive and repulsive IDRs (fig. S8) (*36*). The N-terminal IDR1 was predicted to be attractive, aligning with the data above (Fig. 5A), whereas IDR3 was predicted to be mainly repulsive (fig. S8). These results suggest that an interplay of attractive and repulsive forces within IDRs plays a role in T-tubule formation.

Mammalian HOIP, a catalytic subunit of LUBAC, ubiquitinates itself and other subunits within the complex (*37*, *38*). By analogy, we hypothesized that LUBEL undergoes intra- and/or intermolecular self-ubiquitination. To test this hypothesis, we performed denaturing IP using anti–hemagglutinin (HA) antibody while coexpressing catalytically dead human OTULIN to prevent the degradation of M1-linked ubiquitin chains (*19*, *38*). Immunoblotting with anti–M1-linked ubiquitin antibody revealed high-molecular-weight smear bands (Fig. 5B), suggesting that LUBEL undergoes self-ubiquitination. To identify ubiquitination sites on LUBEL, we immunoprecipitated HA-tagged LUBEL from muscle cells coexpressing HA-LUBEL and catalytically inactive hOTULIN (Cys129→Ala, C129A). Following trypsin digestion, ubiquitinated peptides were enriched using a diGly-specific antibody and then analyzed by liquid chromatography–tandem mass spectrometry (LC-MS/MS). This analysis identified two ubiquitinated Lys residues in LUBEL (Fig. 5B′ and fig. S9). Because the UBA2 domain in LUBEL has been shown to interact with M1-linked ubiquitin chains (*19*), self-ubiquitination may contribute to the multivalency required for the formation of LUBEL puncta (Fig. 5C).

To assess the functional significance of these interactions in T-tubule formation, we conducted rescue experiments using LUBEL constructs lacking either the UBA2 domain or the repulsive IDR (IDR3) (Fig. 5, C and D). In stark contrast to the full-length protein, neither the UBA2-deleted nor IDR3-deleted constructs rescued T-tubule defects in LUBEL mutants (Fig. 5, D′ and D″). These results suggest that attractive interactions mediated by UBA2 and repulsive interactions mediated by the IDR3 together contribute to LUBEL-dependent T-tubule biogenesis.

### LUBEL facilitates Amph-mediated membrane deformation

To evaluate whether LUBEL-mediated M1-linked ubiquitination is sufficient for Amph-dependent membrane deformation, we used HeLa cells as a semireconstituted system by constitutively expressing GFP-tagged *Drosophila* Amph (dmAmph). Stable expression of GFP-dmAmph alone did not induce membrane deformation (Fig. 5E, top). Notably, the transient expression of LUBEL notably enhanced the deformation of Amph-positive structures (Fig. 5E, bottom). Of note, LUBEL and M1-linked ubiquitin chains colocalized with the Amph-positive membranous structures. Furthermore, TEM analysis revealed irregular membrane structures in HeLa cells expressing LUBEL, which were absent in control cells (Fig. 5E′). These findings suggest that LUBEL-containing clusters are responsible for driving Amph-mediated membrane deformation.

### Evolutionary analysis of LUBEL-Amph interaction across animal species

The above results suggest that LUBEL-mediated ubiquitination functions at the early stage of T-tubule biogenesis, similar to caveolins (*11*, *12*). Unlike in *Drosophila*, where T-tubule formation is independent of caveolins, Amph2-mediated T-tubule formation in mammals requires Cav3, which promotes the formation of membrane invaginations (*12*). Caveolin is conserved across metazoans, including nematodes; however, not all caveolin family molecules contribute to membrane deformation (*14*, *39*). Caveolae-forming caveolins, Cav1 and Cav3, are conserved in chordates and vertebrates but absent in other metazoans (Fig. 5F, top). Notably, *Drosophila* lacks caveolin genes entirely (*14*). Evolutionarily, T-tubules emerged before the caveolin-mediated membrane deformation mechanisms (*15*–*17*), indicating the presence of an alternative mechanism in invertebrates, including arthropods. For this reason, we speculate that LUBEL may be this alternative mechanism.

To explore this hypothesis, we conducted an evolutionary analysis of Amph and RNF31, the ortholog of LUBEL and HOIP, across animal species. Both Amph and RNF31 are widely conserved across metazoans. The proline-rich motif in RNF31 is present in a broad range of species, including those within Echinodermata, Ecdysozoa, Spiralia, and Cnidaria, but is absent in Vertebrata, Chordata, and Porifera (Fig. 5F). AlphaFold3 multimer modeling predicted that RNF31 interacts with Amph in *Acanthaster planci* (Ap), *Lingula anatina* (La), and *Nematostella vectensis* (Nv) but not in *Branchiostoma belcheri* (Bb) (fig. S10). To experimentally validate this prediction, we used the Fluoppi system, a fluorescence-based technology that detects protein-protein interaction via puncta formation in mammalian cultured cells. As anticipated, foci were observed in the combination of ApAmph-ApRNF31, LaAmph-LaRNF31, and NvAmph-NvRNF31, whereas foci were hardly detected for BbAmph-BbRNF31 (fig. S11). These findings suggest that the RNF31-Amph interaction arose after the divergence from Porifera and was subsequently lost between the divergence of Echinodermata and the emergence of Vertebrata and Chordata (Fig. 5F). Notably, the evolutionary analysis indicates that the loss of the Amph-RNF31 interaction coincided with the acquisition of Cav1/3 (Fig. 5F). In stark contrast to the results in *Drosophila* (Fig. 1), RNF31 knockdown did not alter T-tubule morphology in zebrafish (fig. S12A). In line with this observation, Cav3 is clearly expressed in both slow and fast muscles, corresponding to the period of T-tubule development in zebrafish (fig. S12B) (*40*, *41*). These data suggest that M1-linked ubiquitination by LUBAC is dispensable for T-tubule formation in vertebrates. On the basis of these findings, we propose that LUBEL-mediated M1-linked ubiquitination plays a role analogous to caveolin in T-tubule biogenesis despite substantial differences in their underlying molecular mechanisms.

## DISCUSSION

**Fig. 6.**
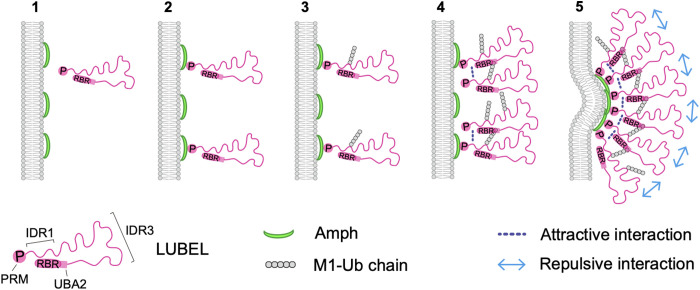
Schematic model of LUBEL-dependent T-tubule biogenesis. Amph initially localizes to the membrane via its amphipathic helix and recruits LUBEL through the interaction between its SH3 domain and the proline-rich motif of LUBEL. LUBEL then self-ubiquitinates and forms multivalent interactions through its IDR1 and UBA2 domains, creating a positive feedback loop that generates LUBEL puncta, which promote membrane tubulation by concentrating Amph and/or inducing membrane curvature.

In this study, we found an unexpected role for M1-linked ubiquitination in T-tubule biogenesis. On the basis of our findings, we propose the following mechanism (Fig. 6): (1) Amph initially localizes to the membrane via its amphipathic helix. (2) LUBEL is recruited to the membrane through the interaction between the SH3 domain of Amph and the proline-rich motif (residues 152 to 157) in LUBEL. (3) LUBEL ubiquitinates itself and its proximity molecules. (4) Multivalent attractive interactions mediated by the IDR1 and UBA2 domain in LUBEL promote further recruitment and autoubiquitination. (5) This positive feedback establishes an amplification loop that drives the formation of punctate structures on the Amph-positive membrane. These puncta facilitate membrane tubulation by increasing the local concentration of Amph and/or by inducing concave membrane deformations through steric pressure.

Membrane localization of Amph is not sufficient to drive membrane tubule extension (Fig. 2). A previous study has shown that, whereas the amphipathic helix of Amph mediates initial membrane binding, tubulation requires high protein densities that enable the formation of oligomeric BAR domain scaffolds (*42*). Given that a minimum threshold of Amph density is necessary for extensive tubulation and that LUBEL clusters are essential for Amph-mediated tubulation by directly interacting with Amph (Fig. 3), it is likely that LUBEL-containing puncta cluster multiple Amph molecules, increase their local concentration on the membrane, and thereby induce membrane deformation.

The initial stage of membrane deformation, where a flat membrane transforms into a tubular shape, is expected to have the highest energy barrier. LUBEL puncta may induce caveola-like shallow membrane deformations in membranes that stimulate Amph polymerization, in addition to increasing Amph concentration, acting as a structural template. Molecular crowding is a plausible mechanism through which the LUBEL clusters generate positive membrane curvature (*43*, *44*). In silico analysis predicted that LUBEL contains both attractive and repulsive IDR (fig. S8). The N terminus proline-rich motif (residues 152 to 157) of LUBEL binds to Amph on the membrane, and cluster formation is possibly mediated by attractive interactions involving IDR1, located near the Amph-binding site, and UBA2. Repulsive interaction mediated by IDR3 within membrane-anchored LUBEL clusters may induce convex membrane bending and enhance both the inherent curvature preference and the polymerization of the Amph BAR domain (Fig. 6). Of note, Amph concentration and the steric pressure are not mutually exclusive—both mechanisms may synergistically contribute to the early stages of T-tubule biogenesis, facilitating further Amph polymerization to drive membrane tubule extension.

Our findings suggest that the LUBEL/RNF31-mediated mechanism represents a caveolin-independent alternative for Amph-mediated tubulation initiation (fig. S13). Evolutionary analysis suggests that the RNF31-dependent membrane deformation mechanism emerged early in animal evolution but was subsequently lost in specific lineages (Fig. 5F). Notably, the loss of the Amph-RNF31 interaction coincided with the emergence of Cav1/3 (Fig. 5F), suggesting a functional transition. On the basis of these findings, we propose that the ancestral RNF31-dependent mechanism was replaced by the caveolin-dependent one during evolution. In vertebrates, the functional specialization of M1-linked ubiquitination in immune regulation may have taken precedence, possibly reflecting the increasing complexity of their immune systems. In contrast, invertebrates have largely retained the RNF31-Amph interaction primarily for its role in membrane deformation processes, such as T-tubule formation.

A ubiquitin reader that recognizes M1-linked ubiquitin chains may play a role in LUBEL-mediated T-tubule biogenesis, similar to other ubiquitin-dependent processes (*45*). However, apart from LUBEL, no ubiquitin-binding domain-containing factors were identified in our RNAi screening (Fig. 1D and data S2). The ubiquitin-binding UBAN domain, found in mammalian ABIN-1/2/3 and NEMO, is known for its high affinity for M1-linked ubiquitin chains and is also present in Optineurin (*46*). In addition, M1-linked ubiquitin-binding NZF and zinc finger domains have been identified in HOIL-1 and A20, respectively (*46*). In contrast, Kenny is currently the only recognized M1-linked ubiquitin-binding protein in *Drosophila* (*31*); however, our results indicate that it is dispensable for T-tubule biogenesis (fig. S3). Further investigation is needed to determine whether a reader associates with the M1-linked ubiquitin chains involved in T-tubule biogenesis.

Structurally, the M1-linked ubiquitin chain resembles the K63-linked chain (*47*). It would be interesting to test whether an artificial LUBEL construct, in which the RBR is replaced by the RING domain of TRAF2 or TRAF6, could function in T-tubule biogenesis. Testing this construct would clarify whether the M1-linked ubiquitin chain can substitute for the K63-linked chain in this process. Furthermore, we cannot rule out the possibility that additional substrates beyond LUBEL are M1-linked ubiquitinated to facilitate T-tubule biogenesis. These LUBEL substrates may include not only proteins but also nonprotein substrates such as lipids and carbohydrates, which have recently been suggested as ubiquitination targets (*48*). Comprehensive identification of such substrates in *Drosophila* will be crucial. Recent advances in ubiquitin-specific proximity-labeling technology offer promising solutions to achieve this goal (*49*, *50*).

Our data demonstrate a previously unidentified concept that ubiquitin-mediated clusters regulate BAR domain proteins. A substantial subset of BAR domain proteins contain scaffolding domains, including SH2, SH3, PDZ, and WW domains (*51*), which can interact with ubiquitin E3 ligases (*8*, *52*, *53*). We propose that, beyond M1-linked ubiquitin chains, other ubiquitin linkage types may also regulate BAR protein functions, broadening our understanding of the molecular mechanisms underlying membrane deformation.

## MATERIALS AND METHODS

### Reagents and antibodies

Streptavidin-HRP (#S911), Phalloidin-Alexa546 (#A22283), anti-HA magnetic beads (#88837, RRID:AB_2861399), glutathione agarose (#16100), and CellMask Orange (#C10045) were purchased from Thermo Fisher Scientific (Waltham, MA, USA). Anti-Dlg1 antibody (clone 4F3, RRID: AB_528203) was obtained from the Developmental Studies Hybridoma Bank (Iowa City, IA, USA). Anti-*Drosophila* Amph antibody was generously provided by C. O’Kane (*17*). Anti-Myc (#562, RRID:AB_591105), anti-HA (#561, RRID:AB_591839), and anti-V5 (#PM003, RRID:AB_592941) antibodies were purchased from MBL (Nagoya, Japan). Anti-HA antibody (clone 3F10, #11867423001, RRID:AB_390918) was obtained from Roche (Basel, Switzerland). Anti–M1-linked ubiquitin antibody was kindly provided by Genentech (South San Francisco, CA, USA) (*54*).

### Generation of transgenic flies

cDNAs encoding the LUBEL and OTULIN variants were integrated into the pUAST-attB vector (RRID:DGRC_1419) using standard molecular cloning techniques. LUBEL and human OTULIN cDNAs were provided by F.I. (*19*). The Amph isoform C cDNA was amplified from larval carcass fillets. The miniTurbo cDNA was amplified from V5-miniTurbo-NES_pcDNA3 (RRID:Addgene_107170) (*22*). Microinjections into the attP landing site stocks were performed by Wellgenetics Inc. (Taipei, Taiwan).

### *Drosophila* strains and maintenance

Flies were maintained under standard conditions at 25°C unless otherwise stated. The *w^1118^* strain was used as a control for LUBEL mutant strains. For muscle-targeted gene expression, Mef2-GAL4 was used. UAS-*LacZ* was used as a control for UAS transgenes. Detailed genotypes are described in table S1. The following fly stocks were used:

1) *y,w*; *P*{*w^+mC^=GAL4-Mef2.R*}3 (Bloomington *Drosophila* Stock Center, BDSC_27390), referred to as DMef2-GAL4;

2) *w*; UAS-V5-miniTurbo (kindly provided by S. Jean, University of Sherbrooke), referred to as UAS-V5-miniTurbo;

3) *w*; *{UAS-Amph^isoform_A^-miniTurbo}attP40* (this study), referred to as UAS-Amph-V5-miniTurbo;

4) *w; P{w[+mC]=UAS-Dcr-2.D}10* (BDSC_24651), referred to as UAS-Dcr2;

5) *w*, *P{PTT-GC}dlg1[YC0005]* (BDSC_50859), referred to as Dlg1-GFP;

6) *w*; *P*{*w^+mC^=UAS-lacZ.B*}*Bg4-1-2* (BDSC_1776), referred to as UAS-LacZ;

7) *y,v; P{TRiP.JF02883}attP2* (BDSC_28048), referred to as UAS-IR-Amph;

8) *w*, *UAS-IR-LUBEL^11321R-2^* (NIG-RNAi_11321R-2), referred to as UAS-IR-LUBEL_NIG;

9) *w*; *UAS-IR-LUBEL^KK106140^* (VDRC_100651), referred to as UAS-IR-LUBEL_KK;

10) *w*; *UAS-IR-LUBEL^GD18055^* (VDRC_18055), referred to as UAS-IR-LUBEL_GD;

11) *y,w; Mi{ET1}LUBEL[MB00197]* (BL_22725), referred to as LUBEL^MI^;

12) *LUBEL^DelR2^* (*19*);

13) *LUBEL^CC/SS^* (*19*);

14) *w*; *{UAS-3xFLAG-hsOTULIN}attP2* (this study), referred to as UAS-hOTULIN;

15) *w*; *{UAS-3xFLAG-hsOTULIN^C129A^}attP2* (this study), referred to as UAS-hOTULIN^C129A^;

16) *w; Rel[E20] e[s]* (BDSC_9457), referred to as Rel[E20];

17) *w; PBac{w[+mC]=PB}key[c02831]* (BDSC_11044), referred to as kenny[c02831];

18) *w*; *{UAS-3xHA-LUBEL}attP2* (this study), referred to as UAS-HA-LUBEL;

19) *w*; *{UAS-3xHA-LUBEL^1-872^}attP2* (this study), referred to as UAS-HA-LUBEL^1-872^;

20) *w*; *{UAS-3xHA-LUBEL^872-2902^}attP2* (this study), referred to as UAS-HA-LUBEL^872-2902^;

21) *w*; *{UAS-miniTurbo-3xHA-LUBEL}attP2* (this study), referred to as UAS-mnTb-LUBEL;

22) *w*; *{UAS-mNeonGreen-3xHA-LUBEL}attP2* (this study), referred to as UAS-mNG-LUBEL;

23) *w*; *{UAS-3xHA-LUBEL*^Δ*2466-2521*^*}attP2* (this study), referred to as UAS-HA-LUBEL^ΔUBA2^;

24) *w*; *{UAS-3xHA-LUBEL^Δ1214-2441^}attP2* (this study), referred to as UAS-HA-LUBEL^ΔIDR^; and

25) *w*; *{UAS-Amph^isoform_C^:3xV5}attP2* (this study), referred to as UAS-Amph-V5.

### RNAi screening

UAS-RNAi lines (data S2) were crossed with *w, Dlg1-GFP*; *Mef2-GAL4, UAS-Dcr2*. The detailed procedures were previously reported (*21*). Briefly, immobilized 3ILs were mounted between a slide glass and a cover glass following the protocol described by Zitserman and Roegiers (*55*). Dlg1-GFP signals in BWM were imaged using an FV3000 confocal microscope (EVIDENT, Tokyo, Japan) through the dorsal cuticle.

### Immunostaining of larval BWMs

Third-instar larvae (3ILs) were dissected in dissection buffer (5 mM Hepes, 128 mM NaCl, 2 mM KCl, 4 mM MgCl_2_, and 36 mM sucrose) on a 35-mm dish coated with silicone elastomer (SYLGARD 184, Dow Corning, #3097358-1004, Midland, MI, USA) using scissors, forceps, and micro pins (Fine Science Tools, #11295-10, #15000-08, #26002-10, Foster City, CA, USA). The larval carcass fillets were fixed with 4% paraformaldehyde (PFA) in phosphate buffer (Nacalai Tesque, #09154-85, Kyoto, Japan) for 20 min at room temperature. The fixed fillets were washed three times with phosphate-buffered saline (PBS) and incubated with the primary antibody diluted in blocking buffer [0.3% bovine serum albumin (BSA) and 0.6% Triton X-100 or 0.1% digitonin in PBS] overnight at room temperature. The fillets were washed three times with PBS and incubated with the secondary antibody diluted in a blocking buffer for 1 hour at room temperature. The fillets were then washed three times with PBS and mounted with FluorSave (Millipore/Merck, Burlington, Massachusetts, USA).

### Confocal fluorescence microscopy

Both live and fixed samples were observed using an FV3000 confocal microscope (EVIDENT, Tokyo, Japan) equipped with a 60× silicone/1.30–NA (numerical aperture) objective lens (EVIDENT, Tokyo, Japan). FLUOVIEW (EVIDENT, Tokyo, Japan) was used for image acquisition, and the exported images were processed and analyzed with ImageJ (NIH, Bethesda, MD, USA).

### Fluorescence recovery after photobleaching

FRAP experiments were performed on an FV3000 confocal microscope (EVIDENT, Tokyo, Japan). Live imaging was conducted on 3IL BWMs expressing mNG-LUBEL. Larval carcasses were mounted in dissection buffer (5 mM Hepes, 128 mM NaCl, 2 mM KCl, 4 mM MgCl_2_, and 36 mM sucrose) and covered with a coverslip for imaging. A defined region of interest (ROI) was photobleached using high-intensity 488-nm laser illumination. Five ROIs were imaged, and fluorescence recovery was monitored over time with low laser power to minimize additional photobleaching. Images were acquired approximately every 2 s.

Fluorescence intensities within the bleached ROI were quantified using ImageJ/Fiji. At each time point, the measured intensity was first normalized by dividing it by the average fluorescence intensity of the entire image. This value was then normalized to the prebleach intensity, which was set to 100%.

### Proximity labeling in larval BWMs and MS analysis of biotinylated peptides

Second or early 3ILs expressing miniTurbo-fused constructs under the control of Mef2-GAL4 were reared on fly media supplemented with 1 mM biotin for 2 days at 25°C. The biotin-fed 3ILs were then dissected to obtain carcass fillets containing BWMs. The fillets were lysed using a guanidine buffer composed of 6 M guanidine hydrochloride, 20 mM tris-HCl, 10 mM tris(2-carboxyethyl)phosphine hydrochloride, 40 mM chloroacetamide, and a protease inhibitor cocktail (Nacalai Tesque, #04080-11). The biotinylated peptides were purified as described previously (*23*). Briefly, the extracted protein solution was digested with trypsin (MS grade, Thermo Fisher Scientific), and biotinylated peptides were enriched using MagCapture HP Tamavidin 2-REV magnetic beads (#133-18611, FUJIFILM Wako, Japan). The resulting samples were then proceeded for MS.

### Mammalian cell culture

HeLa (RRID:CVCL_0030) and Plat-E (RRID:CVCL_Z232) cells were cultured in Dulbecco’s modified Eagle’s medium (DMEM; Nacalai Tesuque, #08458-45) supplemented with 10% fetal bovine serum (FBS) and 1% penicillin-streptomycin. Cells were maintained at 37°C in a humidified environment with 5% CO_2_.

### *Drosophila* S2 cell culture

S2 cells (RRID:CVCL_Z232) were cultured at 25°C in Schneider’s *Drosophila* medium (Thermo Fisher Scientific, #21720-024) supplemented with 10% FBS and penicillin-streptomycin.

### Plasmid transfection

JetPRIME Transfection Reagent (Polyplus-transfection, Illkirch, France) was used for plasmid transfection into HeLa, Plat-E, and S2 cells. Cells were plated in wells or dishes 1 day before transfection to achieve 50 to 70% confluence on the day of transfection. Plasmid DNA was diluted using JetPRIME buffer (1 μg of DNA per 100 μl buffer). Following this, JetPRIME Transfection Reagent was added to the diluted DNA at a ratio of 2:1 (2 μl of JetPRIME for 1 μg of plasmid DNA) and incubated at room temperature for 10 min. The resulting DNA/JetPRIME complexes were then added dropwise to cells in growth medium, and the transfected cells were incubated for 2 days to allow for gene expression.

### Generation of HeLa cells stably expressing dmAmph-EGFP

Plat-E cells were transfected with pMRX-Amph_C-GFP-IRES2-puroR and pVSV-G. The supernatants containing recombinant retrovirus were filtered through a 20-μm filter. HeLa cells were then infected with the retrovirus solution, supplemented with polybrene (8 μg/ml) for 6 hours. Stable transformants were then selected using puromycin (1 μg/ml) for 1 week.

### Immunostaining of HeLa cells

HeLa cells were cultured on 15-mm cover glasses in 12-well plates and fixed with 4% PFA in PBS for 10 min at room temperature. The fixed cells were washed twice with PBS and incubated with the primary antibody diluted in blocking buffer (1% BSA and 0.1% Triton X-100 in PBS) for 1 hour at room temperature. The cells were washed twice again with PBS and incubated with the secondary antibody diluted in a blocking buffer for 1 hour at room temperature. The stained samples were then washed twice with PBS and mounted using FluorSave (Millipore/Merck, Burlington, MA, USA).

### Protein-protein interaction assay using the Fluoppi system

The coding region of the Amph SH3 domains and the RNF31 proline-rich regions from *B. belcheri*, *A. planci*, *L. anatina*, and *N. vectensis* were synthesized by Integrated DNA Technologies Inc. (Coralville, IA, USA). These synthesized DNA fragments were cloned into the pAzamiGreen and pHA-Ash vectors using the EcoRI and BamHI restriction sites. HeLa cells were transfected with pAzamiGreen-RNF31 (proline-rich region) and pHA-Ash-Amph (SH3 domain), followed by immunostaining with anti-HA antibody (3F10). Confocal imaging was performed using an FV3000 microscope (EVIDENT, Tokyo, Japan), and image analysis was conducted using ImageJ Fiji. Each cell was manually cropped and processed using a median filter and the “Subtract Background” function. The resulting single-cell images were converted to binary images using the “Threshold” function. To assess colocalization, binary images from AzamiGreen and HA-Ash channels were multiplied using the ImageCalculator function. The percentage of colocalized dots was then quantified using the “Measure” function. For the Fluoppi analysis (fig. S5), a minimum of 15 cells were analyzed for each combination.

### IP and GST pull-down

S2 cells grown in 6-well plates or 20 3IL carcass fillets were lysed with 200 μl of lysis buffer [1% NP-40, 1 mM EDTA, 20 mM tris-HCl (pH 7.4), and 150 mM NaCl] and centrifuged at 21,130*g* for 10 min at 4°C. For input samples, 20 μl of the supernatant was mixed with 20 μl of 2x sample buffer [4% SDS, 20% glycerol, 125 mM tris-HCl (pH 6.8), 0.001% bromophenol blue, and 200 mM dithiothreitol]. For IP, 150 μl of the supernatant was incubated with 10 μl of anti-HA magnetic beads (Thermo Fisher Scientific, #88837) or GST-fused protein-conjugated glutathione agarose (Thermo Fisher Scientific, #16100) for 90 min at 4°C. For denaturing IP, lysis buffer was supplemented with 1% SDS, and the lysate was subsequently diluted 10-fold with SDS-free lysis buffer before IP. The beads were then washed three times with 500 μl of lysis buffer and eluted with 20 μl of 1x sample buffer by heating at 95°C for 5 min. For MS analysis, 5 μl of anti-HA magnetic beads was used for IP. The samples were then washed and resuspended in 50 mM triethylammonium hydrogen carbonate solution (Fujifilm Wako Chemicals, Osaka, Japan, #206-08381)

### Western blotting

The samples were subjected to SDS–polyacrylamide gel electrophoresis (PAGE) using a precast gel (SuperSep Ace, 5 to 20%, 17 wells, Fujifilm Wako Chemicals, #194-1521). Proteins in the acrylamide gel were transferred onto a polyvinylidene difluoride (PVDF) membrane (Millipore/Merck, Immobilon-PSQ, #ISEQ00010) using transfer buffer (0.1% SDS, 1.2% Tris base, 1.44% glycine, and 20% methanol). The transfer was conducted at a current of 2 A or a voltage of 15 V for 1 hour with the Trans-Blot Turbo transfer system (Bio-Rad, #1704150 J1). Following the transfer, the membrane was blocked [1% BSA, 0.1% Tween 20, and 10 mM tris-HCl (pH 8.0)] for 10 min at room temperature. It was then incubated with the primary antibody diluted in Can Get Signal buffer (Toyobo, #NKB-101, Osaka, Japan) overnight at room temperature. The membrane was washed three times with TBS-T [0.1% Tween 20, 10 mM tris-HCl (pH 8.0), and 150 mM NaCl] and then incubated with an HRP-conjugated or StarBright Blue 700–conjugated secondary antibody diluted in blocking buffer for 1 hour at room temperature. The membrane was washed again three times with TBS-T. Immunoreactive bands were detected using the Clarity Western ECL Substrate (Bio-Rad, Hercules, CA, USA) and ChemiDoc MP Imaging System (Bio-Rad, Hercules, CA, USA). The resulting images were processed using ImageJ.

### MS analysis of coimmunoprecipitated proteins

HA-LUBEL^1-872^ or LacZ (control) was expressed under the control of Mef2-GAL4, and the progenies were raised at 25°C. The 3IL carcass fillet lysates were subjected to anti-HA IP. The immunoprecipitated proteins bound to the beads were washed twice with lysis buffer and then twice with 50 mM triethylammonium bicarbonate. Proteins on the beads were digested by adding 200 ng of trypsin at 37°C overnight. The digested peptides were reduced, alkylated, acidified, desalted using a GL-Tip SDB, and dried. Peptides resuspended in 0.1% trifluoroacetic acid and 3% acetonitrile (ACN) were analyzed by an EASY-nLC 1200 UHPLC-coupled Orbitrap Fusion mass spectrometer (Thermo Fisher Scientific). The peptides were separated on a C18 reversed-phase column (75 μm by 150 mm; Nikkyo Technos) with a linear 4 to 32% ACN gradient for 0 to 100 min with a flow rate of 300 nl/min, followed by an increase to 80% ACN for 10 min and a final hold at 80% ACN for 10 min. Peptides were ionized by electrospray ionization at 2.0 kV. The data-dependent acquisition method was used to acquire MS/MS spectra with a maximum duty cycle of 3 s. The primary MS scan (MS1) was acquired in the Orbitrap at a 120,000 resolution with a maximum injection time of 50 ms. Then, the most abundant ions within a scan range of 375 to 1500 mass/charge ratio (*m/z*) based on the *m/z* signal with charge states of 2 to 7 from that scan were chosen from the MS1 for collision-induced dissociation in the HCD cell and MS2 analysis in the linear ion trap with an AGC target of 1 × 10^4^, an isolation window of 1.6 *m/z*, a maximum injection time of 35 ms, and a normalized collision energy of 30. Dynamic exclusion was set to 20 s. Raw data were directly analyzed against the SwissProt database restricted to *Drosophila melanogaster* using Proteome Discoverer 2.5 (Thermo Fisher Scientific) with the Sequest HT search engine. The search parameters were as follows: trypsin as an enzyme with up to two missed cleavage, precursor mass tolerance of 10 parts per million (ppm), fragment mass tolerance of 0.6 Da, carbamidomethylation of cysteine as a fixed modification, and acetylation of the protein N terminus and oxidation of methionine as variable modifications. Peptides were filtered at a false discovery rate of 1% using the Percolator node. Label-free quantification was performed on the basis of the intensities of precursor ions using the Precursor Ions Quantifier node.

### MS analysis of diglycine-modified peptides

HA-LUBEL was expressed under the control of Mef2-GAL4, and the progenies were raised at 25°C. The 3IL carcass fillet lysates were subjected to anti-HA denaturing IP. The immunoprecipitated proteins bound to the beads were washed twice with lysis buffer and then twice with 50 mM ammonium bicarbonate. Proteins on the beads were digested with 400 ng of trypsin/Lys-C mix (Promega) for 16 hours at 37°C. The digests were reduced, alkylated, diluted 10-fold with HBS [50 mM Hepes-NaOH (pH 7.5) and 150 mM NaCl], and processed using a PTMScan HS Ubiquitin/SUMO Remnant Motif (K-ε-GG) Kit (Cell Signaling Technology). The eluates in 0.1% TFA and 60% ACN were evaporated, desalted, and dried.

LC-MS/MS analysis of the resultant peptides was performed on a nanoElute 2 coupled with a timsTOF HT mass spectrometer (Bruker) as described previously (*56*). The search parameters were as follows: (i) trypsin as an enzyme with up to two missed cleavages; (ii) precursor and fragment mass tolerances of 20 ppm; (iii) carbamidomethylation of cysteine as a fixed modification; and (iv) acetylation of the protein N terminus, oxidation of methionine, and diglycine modification of lysine as variable modifications.

### Quantitative real-time PCR

Total RNA was extracted from 3IL carcasses using TRIzol reagent (Invitrogen, Carlsbad, CA, USA, #15596026) according to the manufacturer’s instructions. Each biological replicate consisted of a pool of three individual larvae, and three independent biological replicates were prepared for each condition. Reverse transcription was performed using the PrimeScript RT reagent Kit with gDNA Eraser (Takara, Shiga, Japan, #RR092S) following the manufacturer’s protocol.

Quantitative real-time polymerase chain reaction (PCR) was performed using the THUNDERBIRD Next SYBR qPCR Mix (Toyobo, Osaka, Japan, #QPX-201) on a QuantStudio 3 Real-Time PCR System (Applied Biosystems, Foster City, CA, USA, #272312924). Each 10-μl reaction contained 1 μl of cDNA, 5 μl of 2× SYBR qPCR Mix, and 0.5 μM each primer. The thermal cycling conditions were as follows: 95°C for 30 s, followed by 40 cycles of 95°C for 5 s and 60°C for 12 s. Melt curve analysis was performed to verify the specificity of amplification. The expression level of LUBEL was normalized to the housekeeping gene Rpl32, and relative expression was calculated using the 2^−ΔΔCt^ method. The primer sequences were as follows:

Rpl32_F, 5′-CGGATCGATATGCTAAGCTGT-3′

Rpl32_R, 5′-CGACGCACTCTGTTGTCG-3′

LUBEL_F, 5′-AGCAGTACTCCAACGAACCG-3′

LUBEL_R, 5′-AGCCTTTCTCATGTGCTGCT-3′

### TEM and FIB-SEM

3ILs were pinned on a silicone elastomer–covered petri dish and dissected directly in a fixative solution containing 2% PFA, 2.5% glutaraldehyde, 150 mM sodium cacodylate, and 5 mM calcium chloride (pH 7.4). The larval carcass fillets were fixed for 2 hours at room temperature and then overnight at 4°C. The dissected fillets were washed with 0.1 M phosphate buffer (pH 7.4), postfixed in 1% osmium tetroxide (OsO_4_) buffered with 0.1 M phosphate buffer for 2 hours, dehydrated through a graded ethanol series, and embedded flat in Epon 812 (TAAB, Aldermaston, UK).

For TEM of BWMs, ultrathin sections (70 nm thick) were mounted on copper grids coated with Formvar (Nisshin EM, Tokyo, Japan) and double stained with uranyl acetate and lead citrate. The samples were then observed using a JEM-1400Flash transmission electron microscope (JEOL, Tokyo, Japan).

FIB-SEM tomography was performed using a Helios NanoLab 660 FIB-SEM (Thermo Fisher Scientific, Waltham, MA, USA) as previously described (*57*). Briefly, serial FIB-SEM images were acquired using Auto Slice and View imaging software (Thermo Fisher Scientific) on the same instrument. Two sets of 500 serial images at 50-nm depth intervals were obtained from wild-type and LUBEL mutant BWMs using a backscattered electron detector at an acceleration voltage of 3.0 kV. After alignment, individual FIB-SEM images were manually segmented to identify target membrane structures using the AMIRA 6.1 reconstruction software (Thermo Fisher Scientific), followed by 3D reconstruction.

For TEM of HeLa cells, a glass coverslip with a grid (GC1300, Matsunami Glass, Osaka, Japan) was coated with carbon using a vacuum evaporator IB-29510VET (JEOL, Tokyo, Japan). HeLa cells stably expressing dmAmph-GFP were cultured on the coated coverslip. The cells were then transfected with either an empty vector or plasmid encoding HA-LUBEL and incubated for 16 hours. Subsequently, the cells were fixed with 2% PFA and 0.5% glutaraldehyde in phosphate buffer (pH 7.4) for 1 hour at room temperature. The positions of HA-LUBEL–transfected cells were identified by an FV3000 confocal microscope (EVIDENT, Tokyo, Japan). After fluorescence microscopy, the cells were further fixed with 2.5% glutaraldehyde in 0.1 M phosphate buffer (pH 7.4) for 2 hours at 4°C, followed by postfixation in 1% osmium tetroxide (OsO_4_) in 0.1 M phosphate buffer for 2 hours. The samples were then dehydrated through a graded ethanol series and embedded flat in Epon 812 (TAAB, Aldermaston, UK). Ultrathin sections were prepared by trimming the same region observed by confocal microscopy. These sections, with a thickness of 70 nm, were mounted on copper grids, double stained with uranyl acetate and lead citrate, and observed using a JEM-1400Flash transmission electron microscope (JEOL, Tokyo, Japan).

### Zebrafish experiment

Wild-type *Danio rerio* (AB strain) were maintained at 28.5°C under standard conditions. Embryos were obtained by natural mating and staged in hours postfertilization (hpf). A morpholino targeting *rnf31* (rnf31-aMO, 5′-ATCAGTGAGAGAGGCCATAGCGC-3′) and a standard control morpholino (control-MO, 5′-CCTCTTACCTCAGTT-3′) were purchased from Gene Tools LLC. The rnf31-aMO sequence was based on a previous study (*58*). Morpholinos were injected at 0.6 ng per embryo together with pcDNA3.1-GFP-CAAX plasmid DNA at the one-cell stage. The CAAX motif, derived from human H-Ras (RKLNPPDESGPGCMSCKCVLS), directs GFP to the plasma membrane. GFP-CAAX is used as a T-tubule marker in a previous study (*40*). At 48 hpf, GFP-CAAX fluorescence images were obtained using an FV3000 confocal microscope (EVIDENT, Tokyo, Japan) equipped with XLUMPlanFL 20X lens (EVIDENT, Tokyo, Japan). For line scan analysis, images were preprocessed in ImageJ by applying Gaussian blur (sigma = 3 pixels) to reduce noise, followed by background subtraction using the “Subtract Background” function (rolling ball radius = 50 pixels) to correct for uneven illumination. Cav3 transcriptional profile is obtained from EMBL-EBI Expression Atlas (https://ebi.ac.uk/gxa/experiments/E-ERAD-475/Results) (*59*)

All zebrafish experiments were conducted in accordance with the institutional and national guidelines and regulations. The study protocol was approved by the Institutional Animal Care and Use Committee of Osaka University (RIMD permit #R02-04). The study was conducted in accordance with the ARRIVE (Animal Research: Reporting of In Vivo Experiments) guidelines.

### Prediction of RNF31-Amph interactions using AlphaFold

Protein structure prediction for *D. melanogaster* (Dm) in Fig. 3G was performed using AlphaFold2 via ColabFold (v1.5.2) on Google Colaboratory (*60*). The publicly available notebook, “AlphaFold2.ipynb,” was used. The multiple sequence alignments were generated using MMseqs2 against the UniRef90 and MGnify databases. Structure prediction was performed using the AlphaFold2_multimer_v3 model with five ensemble predictions.

For the analysis shown in fig. S4, including Dm, structure prediction was performed using AlphaFold3 (*61*) via the AlphaFold server (https://alphafoldserver.com/). The protein sequences used for structure prediction were obtained from publicly available databases. The protein sequences of *A. planci* (Ap), *L. anatina* (La), and *N. vectensis* (Nv), and *B. belcheri* (Bb) were identified via NCBI BLASTp using the protein sequence of Dm as the query. The identifiers of the protein sequences are as follows: DmLUBEL/RNF31 (ID: Q8IPJ3), DmAmph (ID: Q7KLE5), ApRNF31 (ID: XP_022084012.1), ApAmph (ID: XP_022084611.1), LaRNF31 (ID: XP_013410818.1), LaAmph (ID: XP_013391361.1), NvRNF31 (ID: XP_032219182.2), NvAmph (ID: XP_048576432.1), BbRNF31 (ID: XP_019627184.1), and BbAmph (ID: XP_019613513.1).

### Image analyses

For quantification of T-tubules, the middle sections of larval BWMs were imaged using confocal microscopy. Each image had a resolution of 1024 × 1024 pixels, corresponding to a physical size of 70.71 μm. Before analysis, regions surrounding the muscle cells were removed. Image processing and quantification were conducted using ImageJ Fiji (NIH, Bethesda, MD, USA). To minimize noise and background, a median filter background subtraction was applied to each image. The processed images were then converted to binary format and further processed using the “Skeletonize” or “Fractal Box Count…” function. The skeletonization index was defined as the mean intensity of the skeletonized binary images. The ImageJ macro used for the skeletonization analysis is provided below:

showMessage(“Select the folder containing Anti-Dlg1 images”);

openDir = getDirectory(“Choose a Directory”);

list = getFileList(openDir);

Array.show(list);

for (i=0; i<list.length; i++){

open(openDir+list[i]);

run(“Median...”, “radius=2”);

run(“Subtract Background...”, “rolling=20”);

setAutoThreshold(“Huang dark”;

run(“Convert to Mask”);

run(“Skeletonize”);

run(“Measure”);

close();

}

The ImageJ macro used for the fractal box–counting analysis is provided below:

showMessage(“Select the folder containing cropped images”);

openDir = getDirectory(“Choose a Directory”);

list = getFileList(openDir);

Array.show(list);

for (i=0; i<list.length; i++){

open(openDir+list[i]);

run(“Median...”, “radius=2”);

run(“Subtract Background...”, “rolling=20”);

setAutoThreshold(“Huang dark”);

run(“Convert to Mask”);

run(“Fractal Box Count...”, “box=2,3,4,6,8,12,16,32,64 black”);

run(“Close All”);

For the skeletonization and the fractal box–counting analysis (Figs. 1, F and G, 3, B, C, and H, 4C, and 5D, and figs. S2 and S3), two images were acquired from each animal, and their average was used to represent a single biological replicate. At least three biological replicates were analyzed per genotype and time point. In the figures, data points from the same animal are plotted in the same color, and the average value for each animal is indicated by a black open circle. The number of biological replicates, statistical test used (Student’s *t* test or Dunnett’s multiple comparison), and corresponding *P* values are indicated in each figure.

For quantification of LUBEL-positive structures (fig. S6C), images were first subjected to a Gaussian blur filter (σ = 1) to reduce noise. Thresholding was then applied using the IsoData method with the dark background option. The thresholded images were converted to binary masks, and particle analysis was performed to measure dot size and roundness. Particles touching the image border were excluded from the analysis, and summary statistics were obtained. A total of 8917 and 6378 particles were analyzed for RU486 conditions of 1.0 and 0.1 μg/ml, respectively. The results are presented as box plots showing the minimum to maximum values. The ImageJ macro used for the morphology analysis is provided below:

run(“Gaussian Blur...”, “sigma=1”);

setAutoThreshold(“IsoData dark”);

setOption(“BlackBackground”, true);

run(“Convert to Mask”);

run(“Analyze Particles...”, “display exclude summarize”);

For line plot analysis, fluorescence intensity profiles along the selected line were obtained using the “Plot Profile” function in ImageJ Fiji (NIH). The intensity data were normalized in Microsoft Excel by setting the maximum and minimum intensities to 1 and 0, respectively. Graphs were generated using GraphPad Prism (GraphPad Software, Boston, MA, USA).

### Prediction of IDR-IDR interactions

Potential interactions between IDRs were predicted using the FINCHES computational pipeline (*36*). The analysis was conducted with version 1.3 of the program implemented in a Google Colaboratory (Colab) environment (https://colab.research.google.com/drive/1WuzvCnRmOiq4nQFYfEeETSnrACBuv7kG). An Mpipi intermap was generated to visualize the predicted IDR-IDR interaction landscape.

### Statistical analysis

GraphPad Prism 9 software (GraphPad, Prism9 version 9.5.1) was used for statistical analyses. Bars represent mean values. When two genotypes were used in an experiment, Student’s *t* test was used. When more than two genotypes were used in an experiment, one-way analysis of variance (ANOVA) with Dunnett’s multiple comparisons test was used. *P* < 0.05 was regarded as statistically significant.
