# Supplementary material for: Linear ubiquitination triggers Amph-mediated T-tubule biogenesis
Source: Sci Adv. 2026 Jan 7;12(2):eady4934. doi: 10.1126/sciadv.ady4934 (PMC12778051; doi:10.1126/sciadv.ady4934)
Supplement: Supplementary file 1 — Figs. S1 to S13 Legend for table S1 Legends for data S1 to S4 Legends for movies S1 and S2 [file sciadv.ady4934_sm.pdf]

Science Advances
AAAS

Supplementary Materials for

Linear ubiquitination triggers Amph-mediated T-tubule biogenesis

Kohei Kawaguchi *et al.*

Corresponding author: Naonobu Fujita, nafujita@cbc.iir.isct.ac.jp

Sci. Adv. **12**, eady4934 (2026)
DOI: 10.1126/sciadv.ady4934

The PDF file includes:

Figs. S1 to S13
Legend for table S1
Legends for data S1 to S4
Legends for movies S1 and S2

Other Supplementary Material for this manuscript includes the following:

Table S1
Data S1 to S4
Movies S1 and S2

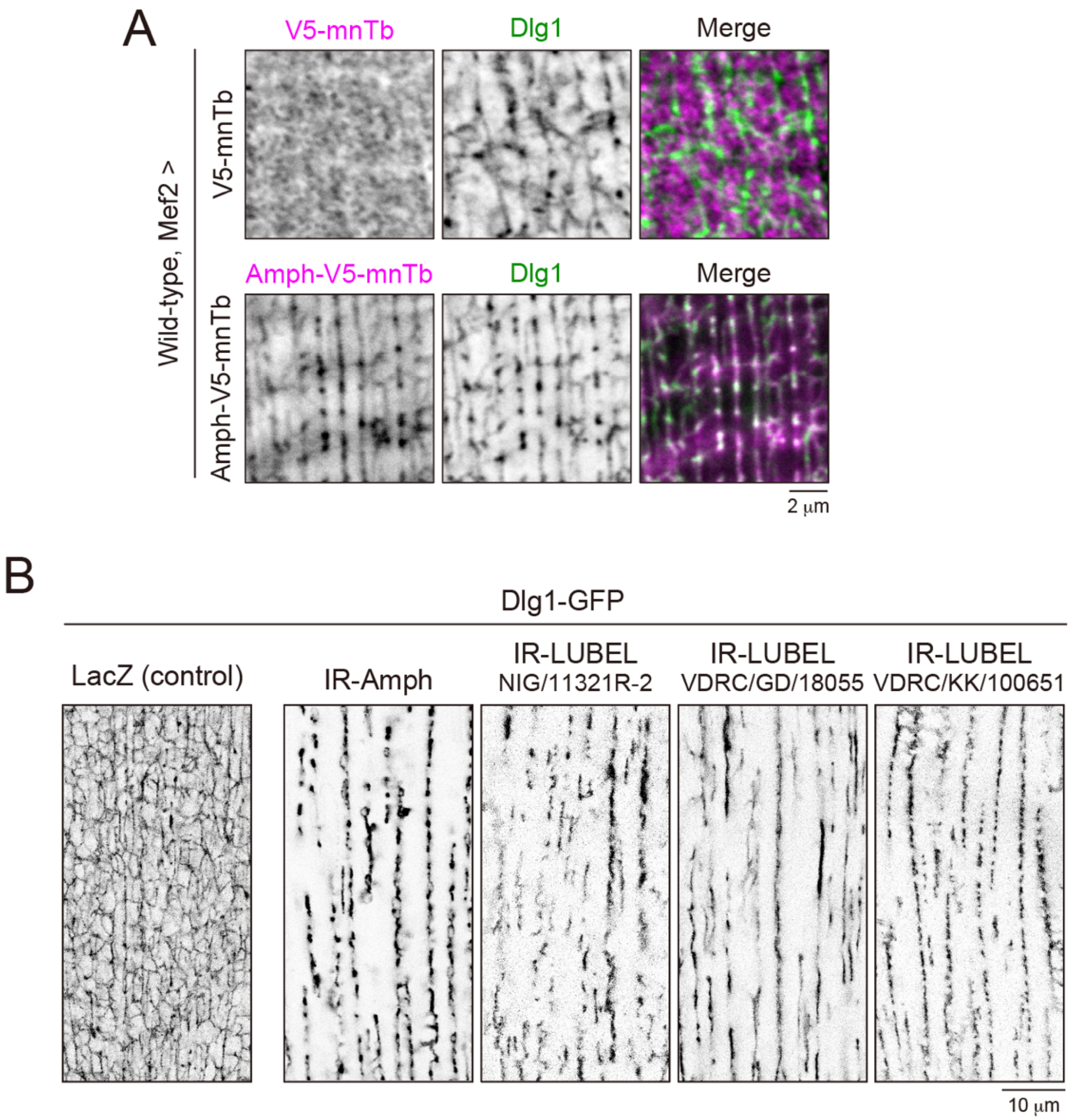

Fig. S1. LUBEL RNAi phenocopies Amph RNAi in T-tubule morphology

(A) Localization of Amph-mnTb or mnTb. Images of anti-Dlg1 and anti-V5 staining in 3IL BWMs. (B) LUBEL or Amph RNAi on Dlg1-GFP-positive T-tubule morphology in 3IL BWMs. Images were acquired through the cuticle using a confocal microscope.

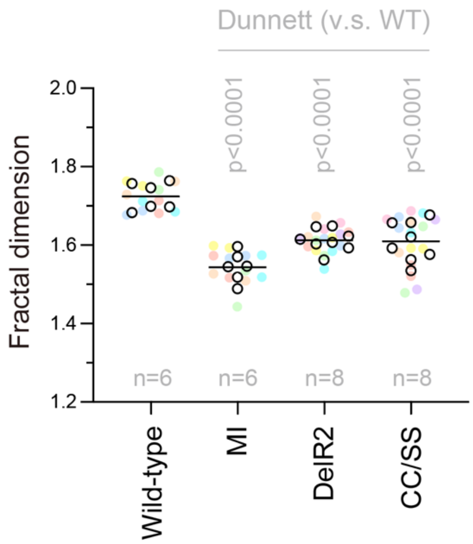

Fig. S2. Fractal box-counting analysis of LUBEL-mutant muscle cells

The data shown in Fig. 1F were analyzed using the fractal box-counting function in ImageJ.

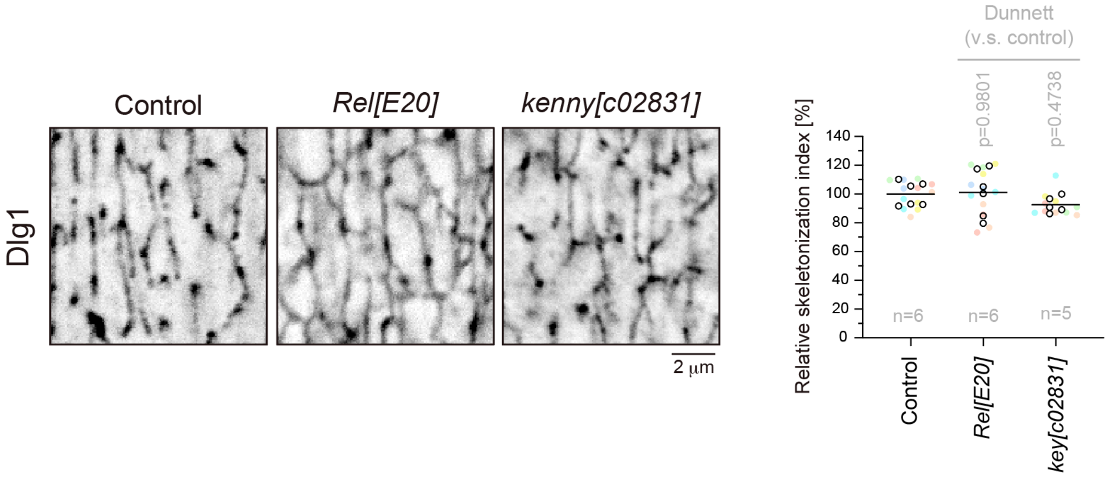

Fig. S3. T-tubule formation is independent of the NF-κB signaling pathway

Images showing anti-Dlg1 staining of 3IL BWMs in the indicated genotypes with the relative skeletonization index quantified.

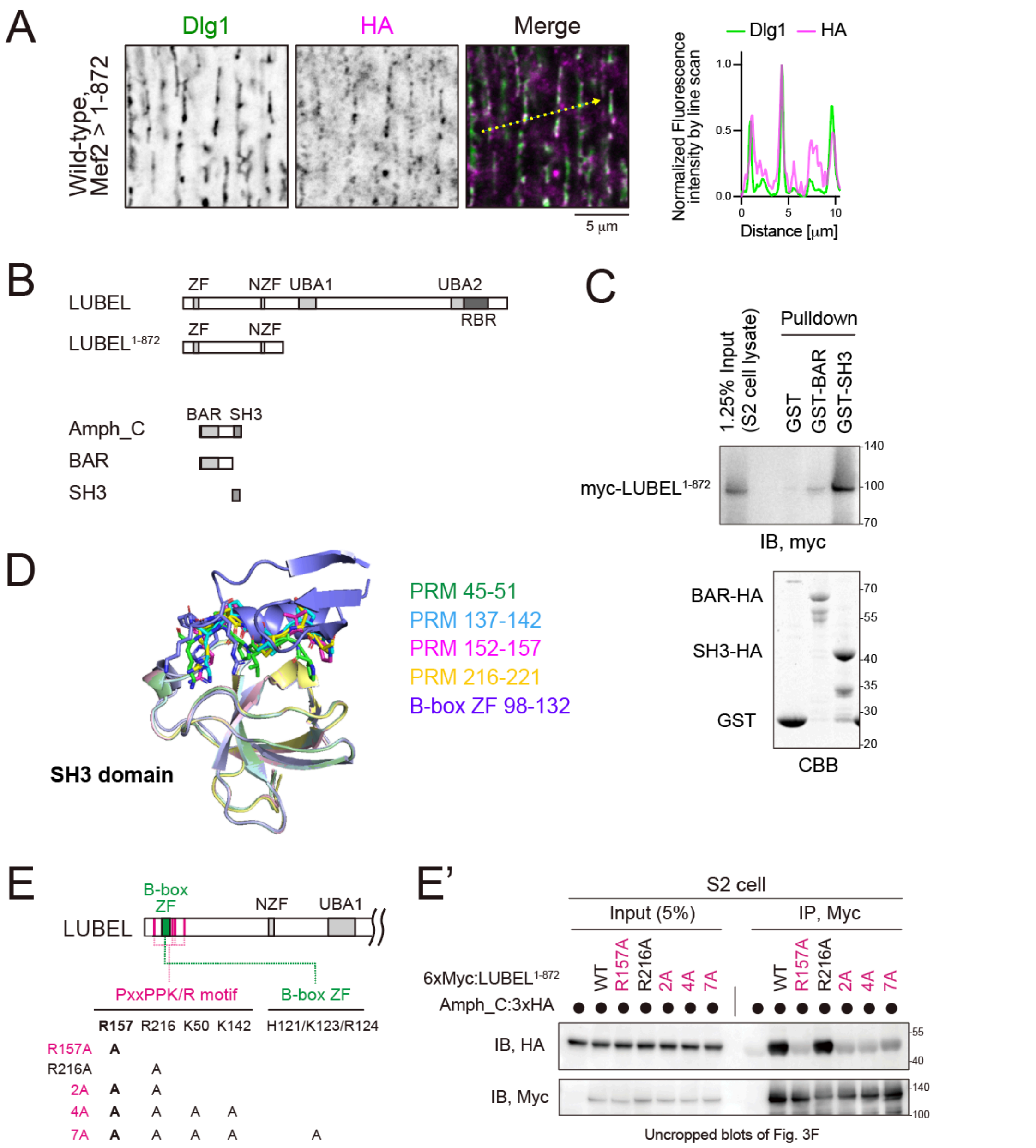

Fig. S4. Characterization of the interaction between LUBEL and Amph

(A) Localization of HA-LUBEL$^{1-872}$. 3IL BWMs expressing HA-LUBEL$^{1-872}$ were stained with anti-Dlg1 and anti-HA antibodies. (B) Schematic representation of LUBEL and Amph truncations. (C) GST pulldown assay of truncated Amph and LUBEL constructs. Myc-LUBEL$^{1-872}$ was expressed in S2 cells. Lysates containing Myc-LUBEL$^{1-872}$ were incubated with beads conjugated to GST-fused truncated Amph isoform C (GST-BAR and GST-SH3). The resultant samples were immunoblotted with anti-Myc antibody or stained with CBB. (D) Structure of the Amph SH3 and LUBEL-N fragment complex using AlphaFold 2. Four proline-rich motifs and a B-box zinc finger are shown. (E) Schematic representation of LUBEL point mutants. (E') Co-IP assay of the LUBEL point mutants and Amph. The Myc-LUBEL$^{1-872}$ fragments harboring the point mutations indicated in (E) were co-expressed with Amph isoform C-HA in S2 cells. Lysates were subjected to anti-Myc IP and immunoblotted for anti-Myc and anti-HA antibodies.

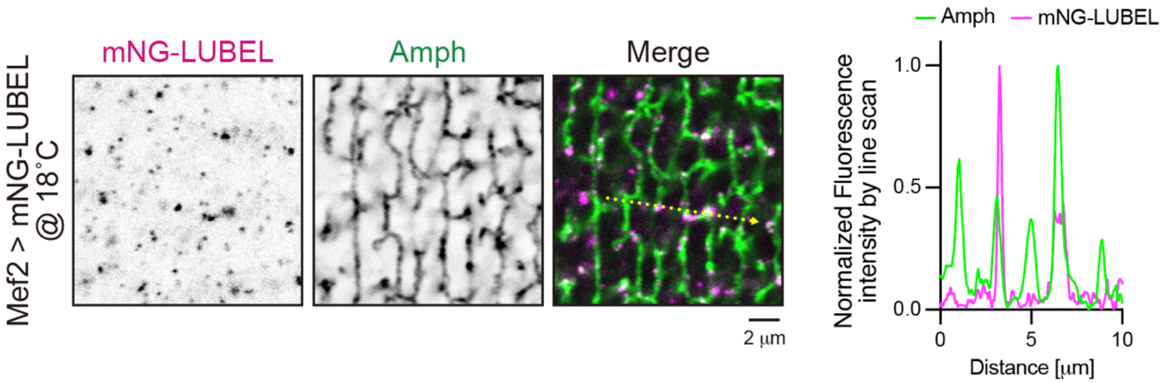

Fig. S5. LUBEL colocalizes with Amph

3IL BWMs expressing mNG-LUBEL were stained with anti-Amph antibody.

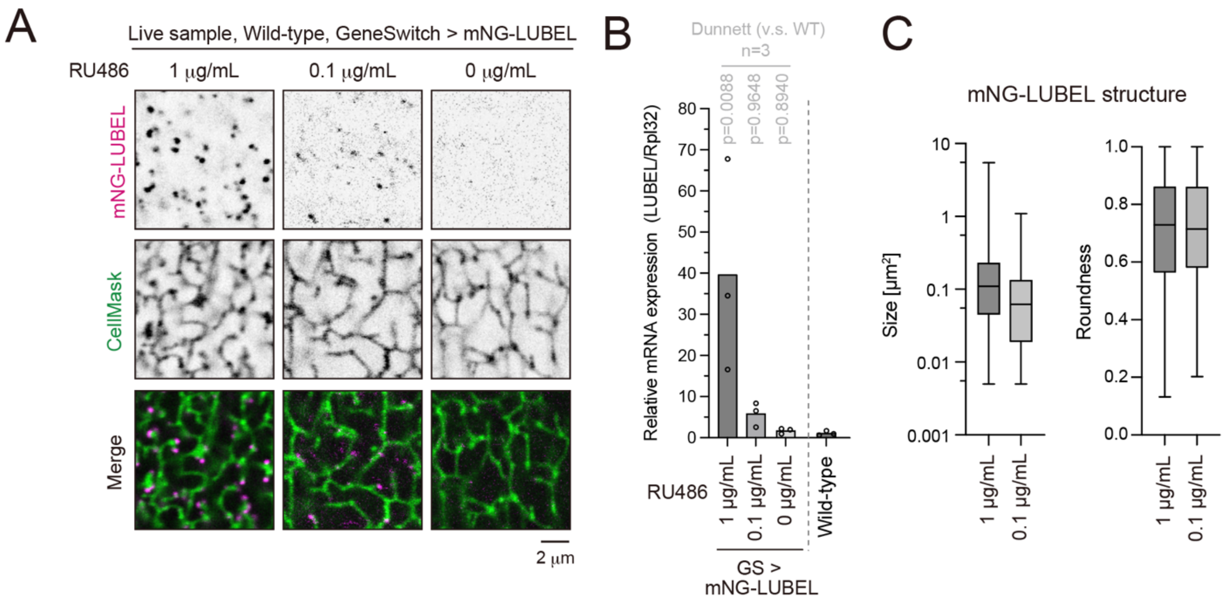

Figure S6. The formation of LUBEL puncta occurs independently of its expression levels
(A) CellMask staining of live wild-type 3IL BWMs expressing mNG-LUBEL under the control of GeneSwitch system. Larvae were treated with RU486 at the indicated concentrations for 24 hours. (B) qRT-PCR analysis of larval carcasses corresponding to the samples shown in (A) and wild-type. (C) Quantification of the size and roundness of mNG-LUBEL structures.

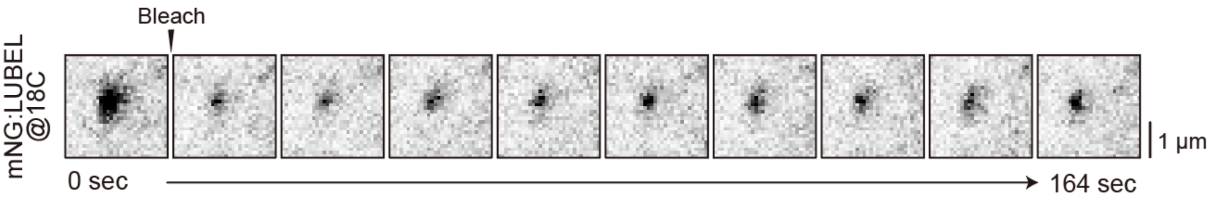

Figure S7. FRAP analysis of LUBEL puncta in LBWM
Time-lapse images of mNG-LUBEL condensates before and after photobleaching.

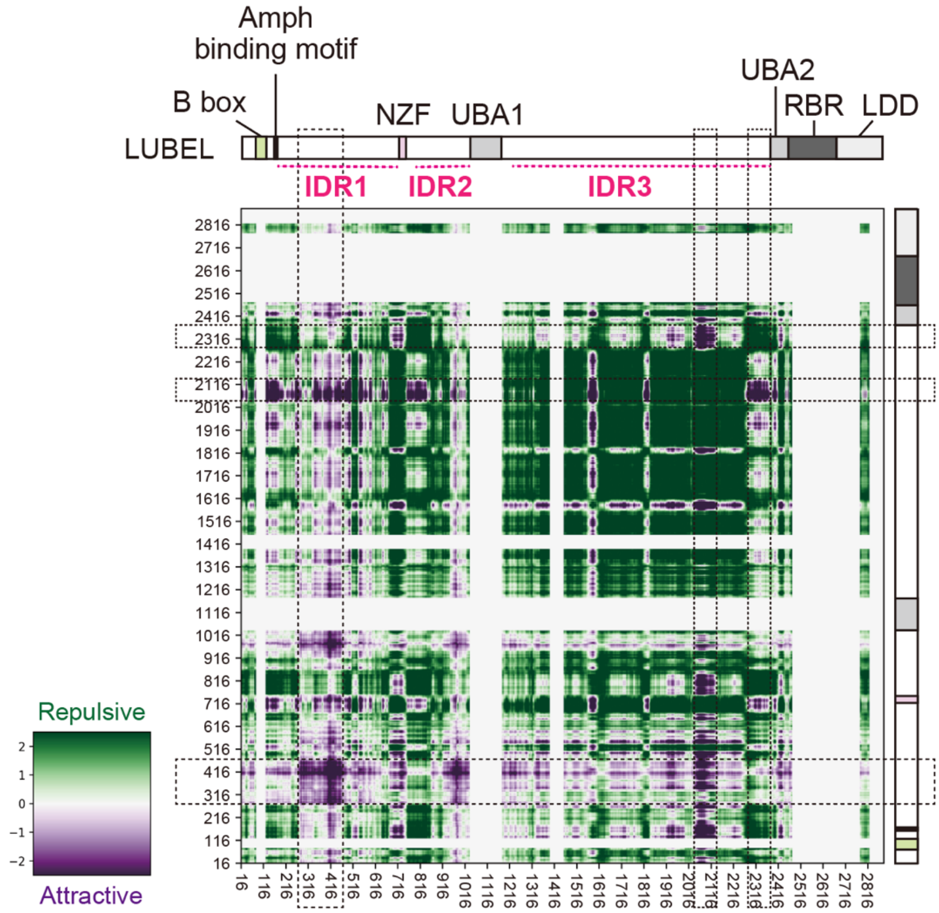

Figure S8. FINCHES intermap for LUBEL.
Green indicates repulsive and purple indicates attractive pairs.

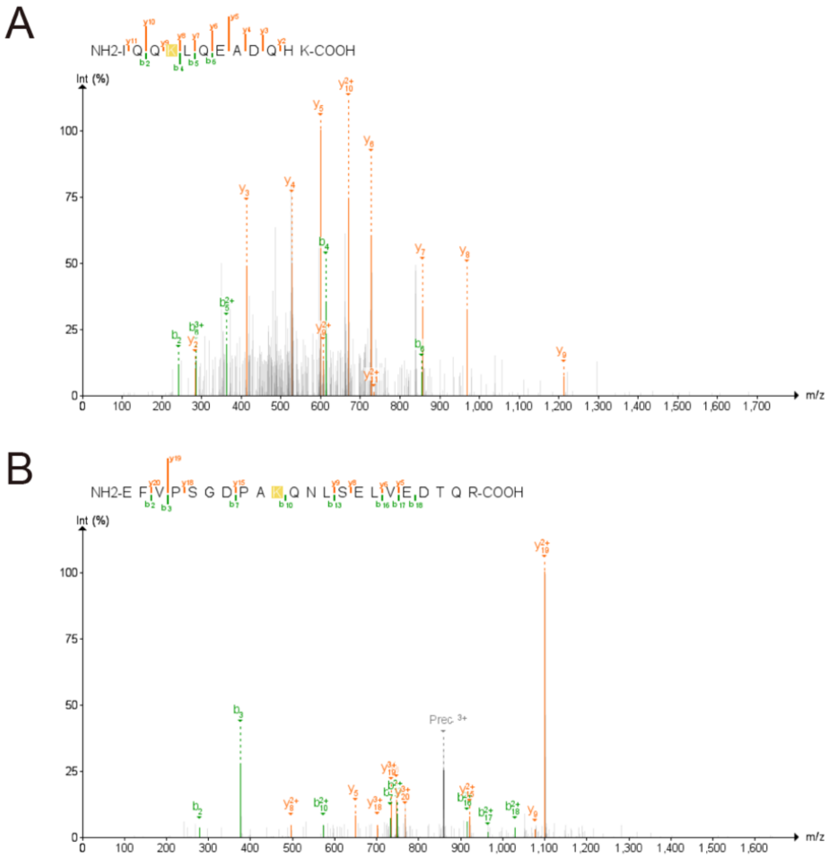

Figure S9. MS/MS spectra of diglycine-modified peptides derived from LUBEL
(A, B) MS/MS spectra of the diglycine-modified peptides identified in the experiment shown in Fig. 5B and 5B’.

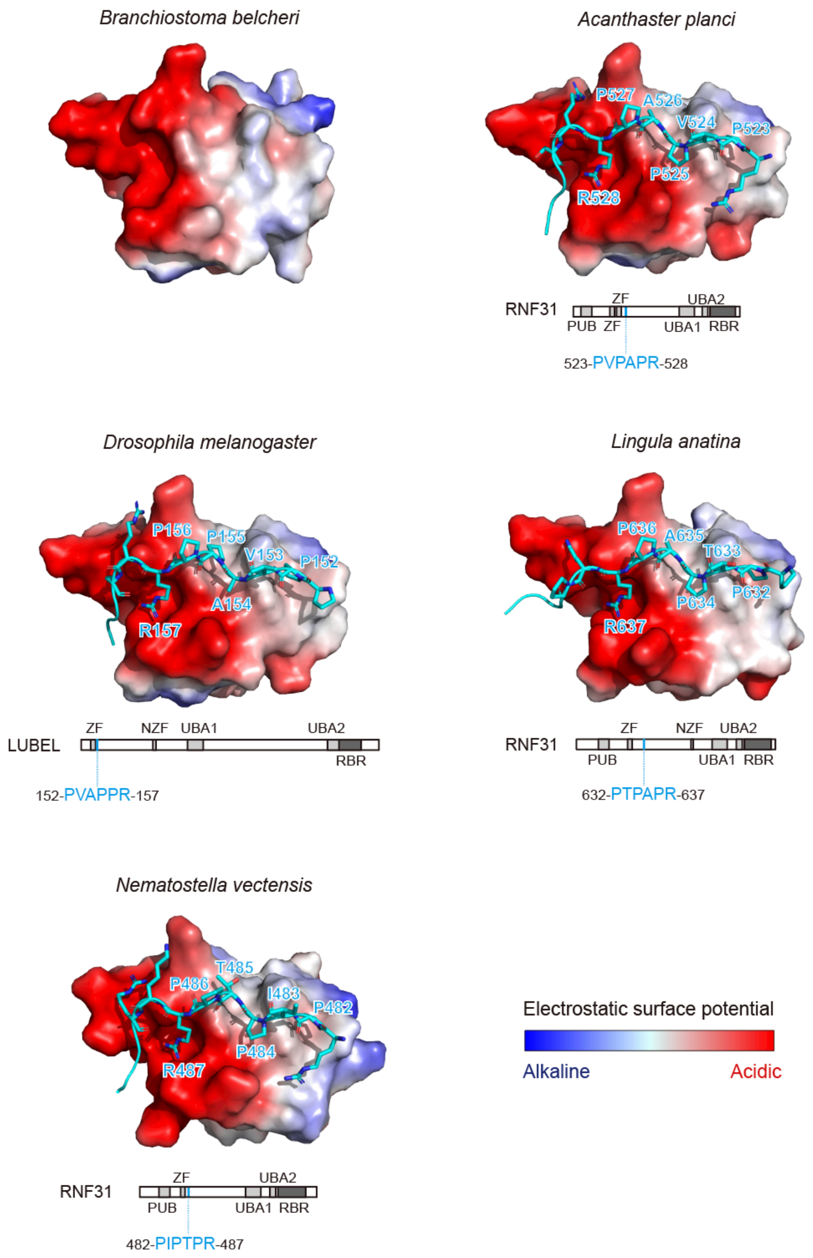

Fig. S10. AlphaFold prediction of the RNF31-Amph interaction

Predicted structures of the Amph SH3–RNF31 complex for each species shown in Figure 5F, generated using AlphaFold3. The Amph SH3 domain is depicted with its surface electrostatic potential, while RNF31/LUBEL is represented as cyan stick models. Below each structure, the domain organization of RNF31/LUBEL from each species is shown, with the Amph-interacting region (proline-rich motif) highlighted in cyan, along with its position and sequence.

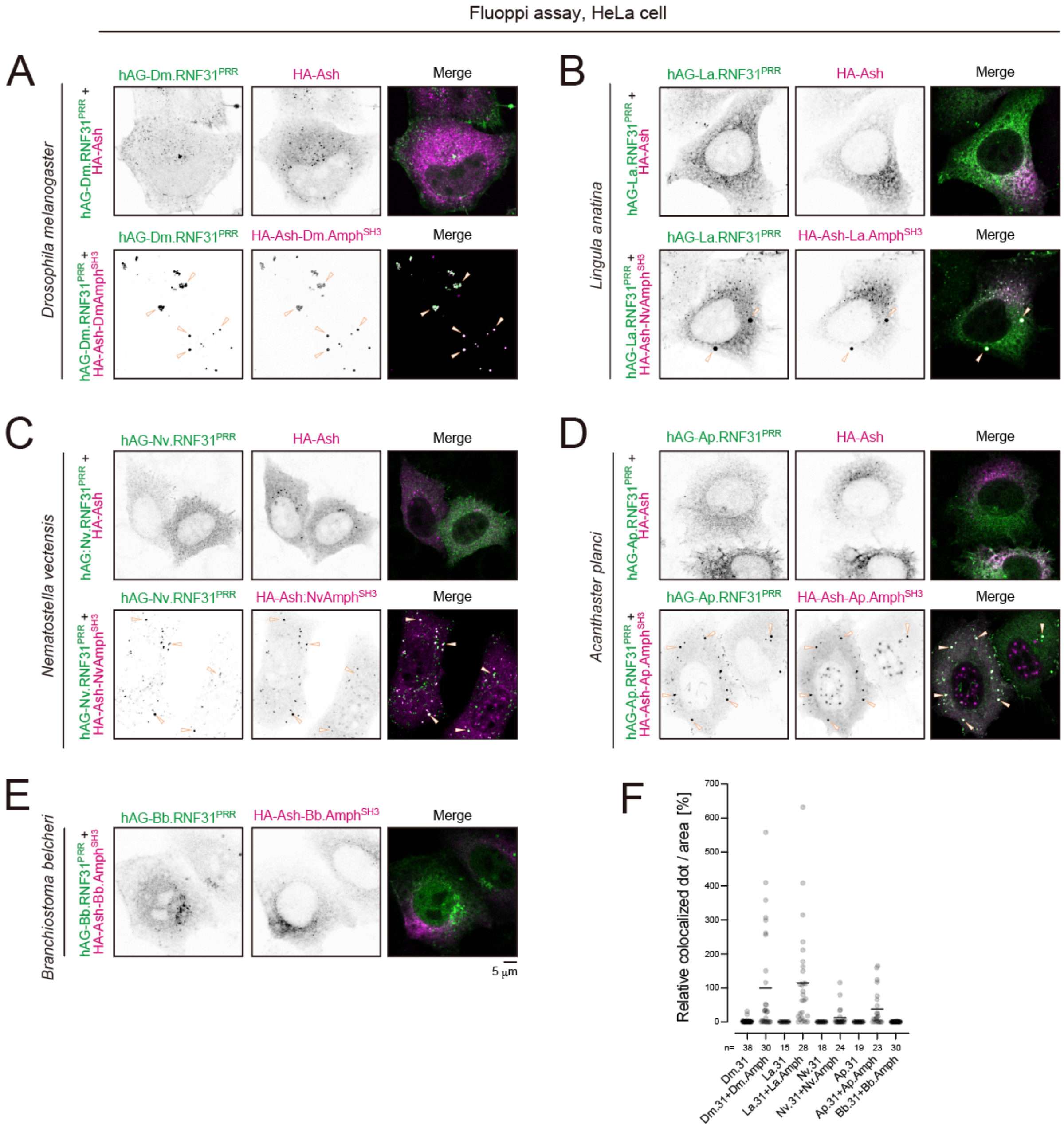

Fig. S11. Protein-protein interaction assay using Fluoppi system

(A-E) Fluoppi analysis of the interaction between RNF31 and Amph in HeLa cells. HeLa cells transiently expressing the indicated constructs from each species listed were stained with anti-HA antibodies. (F) Quantification of foci positive for both RNF31 and Amph.

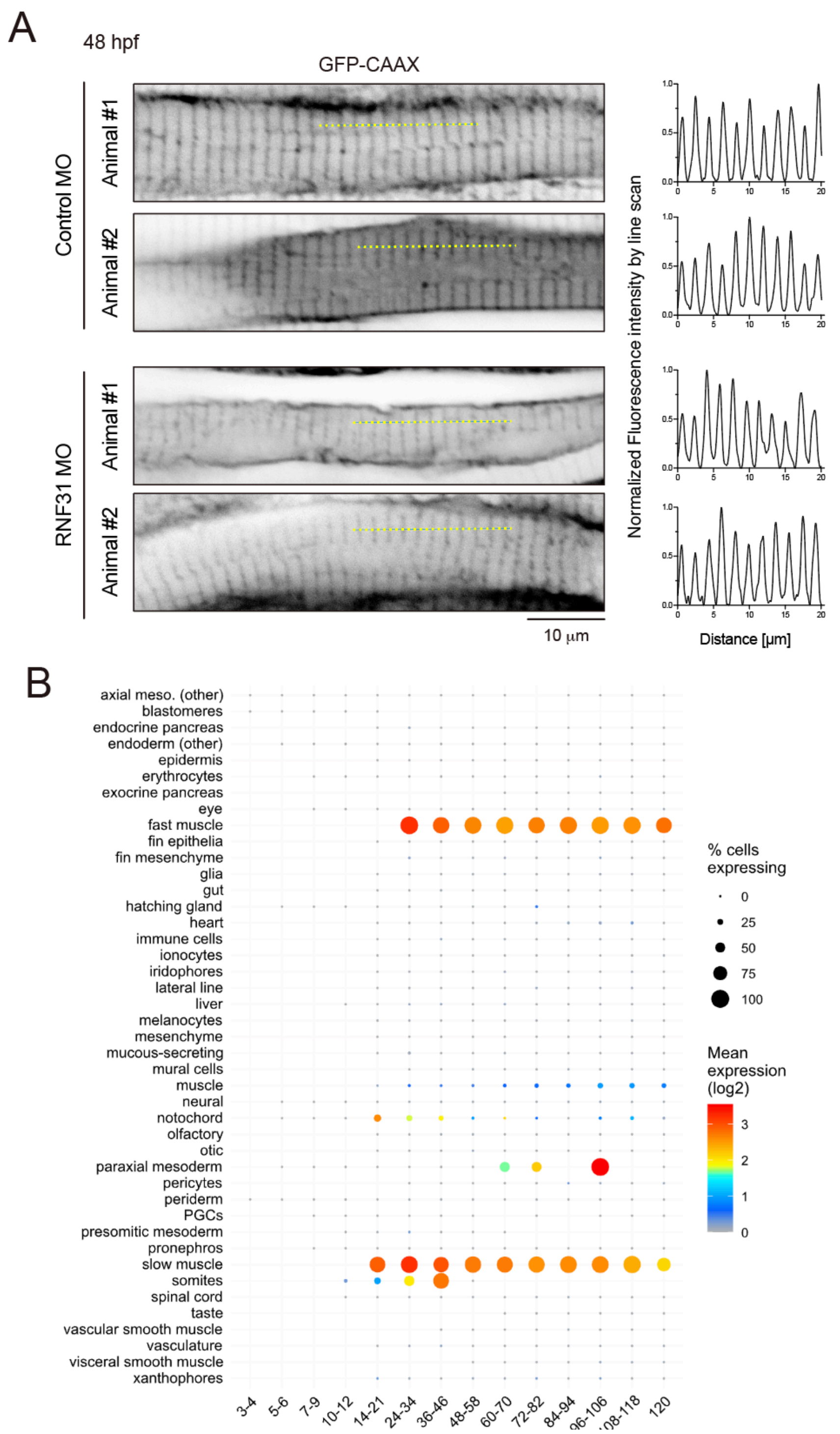

Fig. S12. Knockdown of RNF31 does not disrupt T-tubule morphology in zebrafish

(A) Images showing the T-tubule marker GFP-CAAX in 48 hours post-fertilization (hpf) embryos injected with either control or RNF31-targeting morpholino (MO).

(B) Transcriptional profile of Cav3 during zebrafish development.

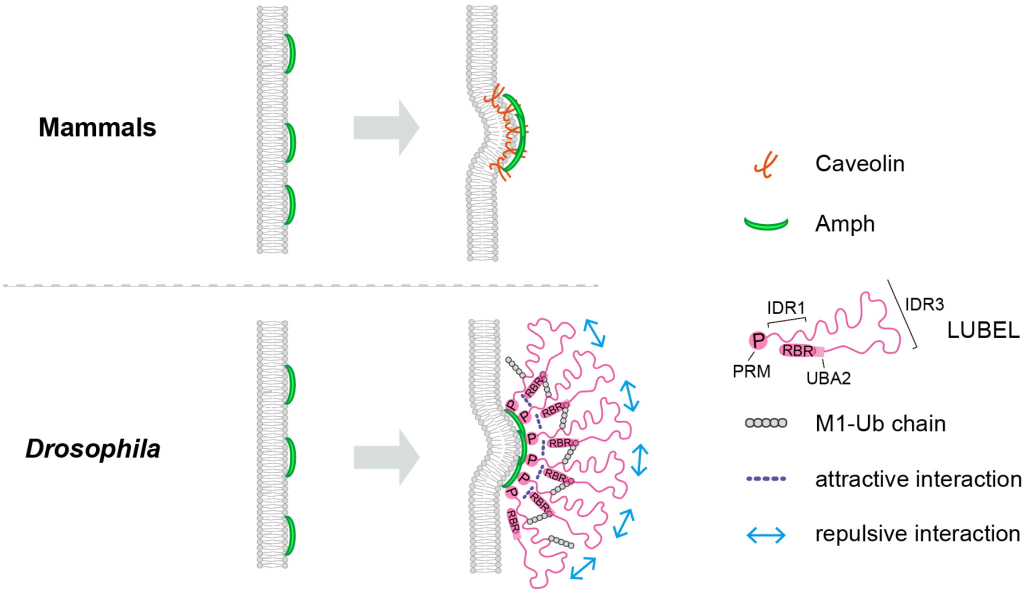

Fig. S13. Schematic models of the early stages of T-tubule biogenesis in mammals and *Drosophila*

Table S1. (separate file)
Detailed Drosophila genotypes used in each figure
Detailed genotypes, stock center identifiers, references, and temperatures are provided.

Data S1. (separate file)
Proximity proteomics of Amph-mnTb
Output table generated by Proteome Discoverer software.

Data S2. (separate file)
RNAi screening of T-tubule formation
Stock center identifiers, CG numbers, and result summaries are shown.

Data S3. (separate file)
IP-MS of HA-LUBEL$^{1\text{-}872}$
Output table generated by Proteome Discoverer software.

Data S4. (separate file)
Proximity proteomics of mnTb-LUBEL
Output table generated by Proteome Discoverer software.

Movie S1
Segmentation and 3D reconstitutions of T-tubules in wild-type larval BWMs, derived from FIB-SEM stacks.

Movie S2
Segmentation and 3D reconstitutions of T-tubule-related membrane structures in *LUBEL*$^{CC/SS}$ mutant larval BWMs, derived from FIB-SEM stacks.
